# Vector-borne zoonotic bacteria in Tunisia: from fragmented data to an integrated One Health surveillance framework

**DOI:** 10.3389/fmicb.2026.1951493

**Published:** 2026-10-12

**Authors:** Mourad Ben Said, Rachid Selmi, Hanène Belkahia

**Affiliations:** 1 Department of Basic Sciences, Higher Institute of Biotechnology of Sidi Thabet, University of Manouba, Manouba, Tunisia; 2 Laboratory of Parasitology, National School of Veterinary Medicine of Sidi Thabet, University of Manouba, Manouba, Tunisia; 3 Laboratory of Microbiology, National School of Veterinary Medicine of Sidi Thabet, University of Manouba, Manouba, Tunisia

**Keywords:** multiple interfaces, One Health, surveillance, Tunisia, vector-borne bacteria, zoonoses

## Abstract

Vector-borne and vector-associated bacterial zoonoses represent an important component of infectious disease epidemiology in Tunisia. This review synthesizes available evidence on major bacterial groups, including *Rickettsia*, *Bartonella*, *Anaplasma*, *Ehrlichia*, *Borrelia*, *Coxiella burnetii*, and other arthropod-associated bacteria, using a One Health framework integrating human, animal, vector, wildlife, and environmental compartments. The available evidence reveals heterogeneous levels of knowledge across bacterial taxa. *Rickettsia* represents the most established tick-associated zoonotic system, with *Rickettsia conorii* as the main confirmed human pathogen, while additional species have been detected mainly in arthropods or animal-associated settings. *Bartonella* has a significant clinical association with blood culture-negative endocarditis, whereas other bacterial groups show predominantly animal- or vector-associated evidence. *Coxiella burnetii* remains a major zoonotic concern linked to livestock, camels, reproductive materials, and environmental exposure. For *Borrelia*, *Anaplasma*, and *Ehrlichia*, substantial evidence of circulation exists in vectors and animals, but human clinical relevance remains incompletely characterized. Across bacterial groups, a recurrent limitation is the imbalance between molecular detection in vectors or animal hosts and confirmed evidence of transmission or human disease. Detection of bacterial DNA should therefore be interpreted cautiously and not considered equivalent to infection, reservoir competence, or vectorial transmission. Additional arthropod-associated bacteria identified through microbiome studies require further investigation to clarify their ecological significance. The Tunisian evidence base demonstrates active circulation of diverse vector-associated bacteria but remains fragmented across clinical, veterinary, entomological, and ecological domains. Strengthening integrated One Health surveillance through coordinated human diagnosis, animal sentinel systems, vector monitoring, environmental investigations, and advanced molecular approaches is essential to better assess zoonotic risks in Tunisia and the wider North African-Mediterranean region.

## 1 Introduction

Vector-borne and vector-associated bacterial zoonoses represent an important but still insufficiently integrated component of infectious disease surveillance in Tunisia. The country occupies a strategic biogeographical position at the interface between the Mediterranean basin, North Africa and the Sahara, with diverse ecological zones ranging from humid northern forests to semi-arid livestock areas and Saharan camel-rearing systems. This environmental heterogeneity supports a wide diversity of arthropod vectors, including ticks and fleas, as well as multiple domestic and wildlife hosts that may contribute to bacterial circulation (Sarih et al., 2005; Bouattour et al., 2010; Balti et al., 2021; Baede et al., 2024; Selmi et al., 2024a,b).

During the last three decades, Tunisian studies have documented several bacterial groups of zoonotic or suspected zoonotic relevance, including *Rickettsia*, *Bartonella*, *Anaplasma*, *Ehrlichia*, *Borrelia*, and *Coxiella* (Letaief et al., 1995; Ghorbel et al., 1994; Znazen et al., 2005; Ben Arab et al., 2008; M’ghirbi et al., 2012; Ben Said et al., 2016; Barkallah et al., 2018; Gharbi et al., 2024). More recently, molecular and microbiome-based studies have expanded this landscape by revealing additional vector-associated bacteria, including tick endosymbionts, opportunistic bacterial genera and emerging or poorly characterized taxa such as *Francisella*-like endosymbionts, *Midichloria mitochondrii* and *Rickettsiella* spp. (Benyedem et al., 2022; Baede et al., 2024; Kratou et al., 2025a,b). These advances have substantially improved knowledge of bacterial diversity in Tunisian vectors and hosts.

However, the available evidence remains fragmented. Human data are often limited to specific clinical syndromes or hospital-based studies, such as Mediterranean spotted fever, blood culture-negative endocarditis, Q fever or suspected Lyme borreliosis (Znazen et al., 2005; Ben Arab et al., 2008; Hammami et al., 2013; Mahfoudhi et al., 2015; Lamloumi et al., 2024). Veterinary and entomological studies, in contrast, have generated extensive data from dogs, cats, ruminants, horses, dromedaries, wildlife, ticks and fleas, but these findings are rarely connected to simultaneous human surveillance (Belkhiria et al., 2017; Zouari et al., 2017; Selmi et al., 2018; Selmi et al., 2020a,b,c; Selmi et al., 2021a,b; ElHamdi et al., 2022). As a result, Tunisia has accumulated valuable but compartmentalized evidence, with limited integration between human medicine, veterinary medicine, entomology, wildlife ecology, environmental exposure, and public health decision-making.

This fragmentation is particularly important because the epidemiological meaning of bacterial detection differs widely between compartments. A bacterium detected in a human clinical sample has different public health significance from the same bacterium detected in a tick, a wildlife host, a microbiome dataset, or seropositive animal. Molecular detection in vectors does not necessarily prove vector competence. Animal positivity does not automatically demonstrate reservoir competence, and microbiome signals should not be interpreted as evidence of zoonotic threats. However, these findings should be interpreted according to several clinical and epidemiological supports. This distinction is essential for agents such as *Anaplasma* spp., which are strongly documented in animals and ticks but not yet confirmed in humans in Tunisia. Besides, tick-associated endosymbionts, such as *Francisella*-like organisms and *Midichloria*, showed a primarily ecological rather than clinical relevance (Belkahia et al., 2015; Ben Said et al., 2017a,b; Benyedem et al., 2022; M’ghirbi et al., 2022; Kratou et al., 2025b).

A One Health approach is highly relevant for assessing vector-borne bacterial zoonoses in Tunisia where multiple interfaces coexist, including dog-tick-human, cat/dog-flea-human, livestock-tick-farmer, camel-*Hyalomma*-human, *Ixodes*-human-wildlife, and tick-microbiome systems.

The present review aims to provide a critical synthesis of vector-borne and vector-associated zoonotic bacteria reported in Tunisia, with emphasis on current evidence, interpretation limits and One Health perspectives. Rather than simply listing reported pathogens, the review evaluates what Tunisian data actually demonstrate, what remains uncertain, and how existing findings could be translated into integrated surveillance and public health actions.

## 2 Methodological approach and one health analytical framework

This review was designed as a structured critical narrative synthesis of vector-borne and vector-associated bacterial zoonoses reported in Tunisia. Because the available studies differed substantially in bacterial targets, host species, arthropod vectors, ecological settings, sample types, diagnostic methods and study designs, no meta-analysis or pooled prevalence estimate was attempted. Instead, the review used an evidence-mapping approach adapted to a One Health risk-interpretation framework, with emphasis on transparency, diagnostic certainty, compartmental convergence and cautious interpretation of heterogeneous evidence (Page et al., 2021; Tricco et al., 2018; FAO, WHO, and WOAH, 2019, 2020).

Literature searches were conducted in PubMed/MEDLINE, Scopus, Web of Science Core Collection, and Google Scholar, and were complemented by searches in major publisher platforms, including ScienceDirect, SpringerLink, Wiley Online Library, Taylor & Francis Online, MDPI and Frontiers. Additional records were identified through backward and forward citation checking, local Tunisian or regional journals, conference abstracts, theses and author-held bibliographic files when they contained usable primary or contextual evidence. The overall study selection process is summarized in Figure 1. The final search was completed on 1 June 2026. No lower date limit was imposed, and all retrievable records published or available up to that date were considered. English and French records were included; records in other languages were considered only when sufficient bibliographic and methodological information could be reliably extracted. The complete search structure, sources and eligibility criteria are provided in Supplementary Table 1.

**FIGURE 1:**
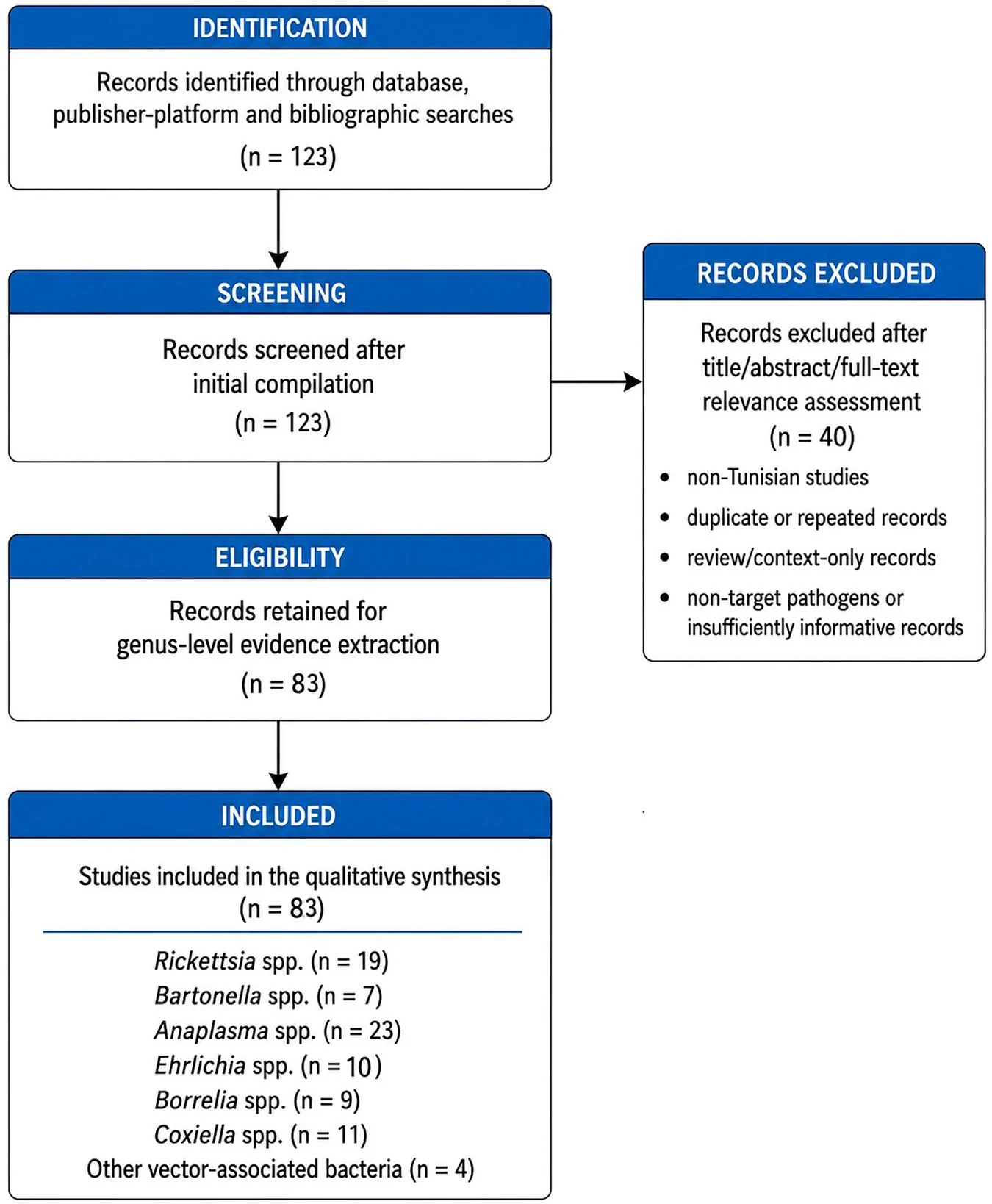
PRISMA-inspired flow diagram summarizing the study selection process for the narrative One Health evidence synthesis of vector-borne and vector-associated bacterial zoonoses in Tunisia. A total of 123 records were identified through database searches (PubMed/MEDLINE, Scopus, Web of Science, Google Scholar), publisher platforms, and additional bibliographic sources. After removal of non-Tunisian studies, duplicates, review/context-only records, non-target pathogens, and insufficiently informative reports, records were screened for eligibility. Following full relevance assessment, 83 records were retained for qualitative evidence synthesis and genus-level extraction. These records were categorized into *Rickettsia* spp. (*n* = 19), *Bartonella* spp. (*n* = 7), *Anaplasma* spp. (*n* = 23), *Ehrlichia* spp. (*n* = 10), *Borrelia* spp. (*n* = 9), *Coxiella burnetii* and related organisms (*n* = 11), and other vector-associated bacteria (*n* = 4). Counts reflect genus-level evidence mapping. Individual studies may contribute evidence to multiple bacterial categories; therefore, genus-level totals are non-exclusive and should be interpreted as overlapping evidence contributions.

Search strings combined country terms with bacterial, disease, vector and host terms. The country terms were Tunisia OR Tunisian. The main genus-level and disease terms were *Rickettsia*, rickettsiosis, Mediterranean spotted fever, *Bartonella*, cat-scratch disease, endocarditis, *Anaplasma*, anaplasmosis, *Ehrlichia*, ehrlichiosis, *Borrelia*, Lyme, relapsing fever, *Coxiella*, Q fever, *Francisella*, *Midichloria*, *Rickettsiella*, *Mycoplasma*, tick microbiome and endosymbiont. These terms were combined with vector and host terms including tick, ticks, flea, fleas, ectoparasite, arthropod, *Ixodes*, *Rhipicephalus*, *Hyalomma*, *Ornithodoros*, *Ctenocephalides*, camel, dromedary, dog, cat, livestock, small ruminant, cattle, horse, wildlife, rodent, hedgehog, and fox. Equivalent French terms were used when relevant.

Records were eligible when they reported original or interpretable evidence on zoonotic, suspected zoonotic, veterinary, vector-associated, wildlife-associated, environmental or microbiome-related bacteria in Tunisia. Eligible evidence included human clinical studies, case reports, animal surveys, vector or ectoparasite screening studies, wildlife studies, environmental or food-chain investigations, microbiome studies, conference abstracts and theses when sufficient methodological details were available. Review articles were not used as primary evidence for evidence grading, but were retained when they provided historical, contextual or methodological information. Records were excluded from the Tunisian evidence synthesis when they were not conducted in Tunisia, addressed viral, parasitic, fungal or non-target bacterial diseases only, provided no usable diagnostic or methodological information, consisted only of inaccessible webpage records, or duplicated a study already extracted. Duplicate records, accepted manuscripts and repeated uploads were merged, and the most complete version was retained.

The evidence was organized into seven bacterial categories: *Rickettsia* spp., *Bartonella* spp., *Anaplasma* spp., *Ehrlichia* spp., *Borrelia* spp., *Coxiella burnetii*/*Coxiella*-like organisms and other vector-associated bacteria. Eligible records were screened, assessed for relevance, and categorized according to bacterial group and One Health compartment. The overall study selection and screening process are summarized in Figure 1, while the detailed distribution of included records among bacterial categories is provided in Supplementary Table 2. Because some studies reported evidence for more than one bacterial genus, genus-level categories represent overlapping contributions and should not be interpreted as independent study totals.

Data extracted from eligible studies included the bacterial taxon or genotype, study type, host or patient group, vector species when applicable, sample type, geographic location, ecological setting, diagnostic approach, molecular targets or serological methods, sequencing or phylogenetic analyses when available, detection rates or seroprevalence estimates, and relevance for One Health surveillance. Findings were interpreted according to the compartment in which they were generated: humans, dogs and cats, livestock, dromedary camels, wildlife, vectors or ectoparasites, environmental or food-chain sources, and microbiome/endosymbiont datasets.

Evidence strength was assessed using a structured qualitative decision framework rather than study counts alone. The classification considered study independence, reproducibility, diagnostic robustness, taxonomic resolution, clinical or epidemiological relevance, One Health compartment coverage, ecological plausibility, geographical representativeness, sampling quality and the quality of negative evidence. Strong evidence required convergent support from robust diagnostic confirmation and/or repeated, clinically relevant or cross-compartmental findings. Moderate evidence indicated credible but incomplete evidence, usually with reliable detection but limited clinical, geographical or compartmental support. Limited evidence indicated isolated, indirect, weakly resolved or microbiome-only signals. Evidence was considered absent or not documented when no relevant Tunisian evidence was identified for a given compartment, or when only well-defined negative studies were available. The decision framework is provided in Supplementary Table 3.

Diagnostic evidence was weighted according to biological and epidemiological certainty. Culture, validated PCR/qPCR, sequencing, multilocus typing and phylogenetic characterization were weighted more strongly than serology, especially when they enabled species- or genotype-level identification. Serological evidence was interpreted cautiously because it may indicate previous exposure rather than active infection, shedding or species-level attribution, and because cross-reactivity may occur in several bacterial groups. In humans, serology was considered more informative when associated with compatible clinical syndromes, confirmatory testing, paired serology, or repeated hospital-based observations. In animals, seropositivity was interpreted mainly as exposure or sentinel evidence rather than proof of active infection, reservoir competence or zoonotic transmission. Microbiome detections and 16S rRNA-based signals were interpreted as ecological or endosymbiotic evidence unless supported by species-level confirmation and independent clinical or epidemiological data. The diagnostic weighting and interpretive rules are summarized in Supplementary Table 4.

## 3 Current evidence by bacterial group

Available Tunisian studies reveal a heterogeneous evidence base across bacterial groups, with marked differences according to human, animal, wildlife and vector-associated available data. These differences are summarized visually in Figures 2, 3, which map the relative strength of evidence across One Health compartments for the main bacterial groups considered in this review. Taxon-level evidence for *Rickettsia*, *Bartonella*, *Anaplasma* and *Ehrlichia* spp. is summarized in Table 1, while corresponding critical gaps and surveillance priorities are presented in Table 2. Evidence for *Borrelia* spp., *Coxiella* spp. and other vector-associated bacteria is summarized in Table 3, with related gaps and priorities presented in Table 4. For each genus or bacterial category, the evidence is therefore interpreted according to the type of host investigated, the arthropod vectors involved, the diagnostic methods used, and the strength of the One Health link. Particular caution is needed when interpreting molecular detection in vectors, as the presence of bacterial DNA does not necessarily imply active infection, vector competence or confirmed zoonotic transmission.

**FIGURE 2:**
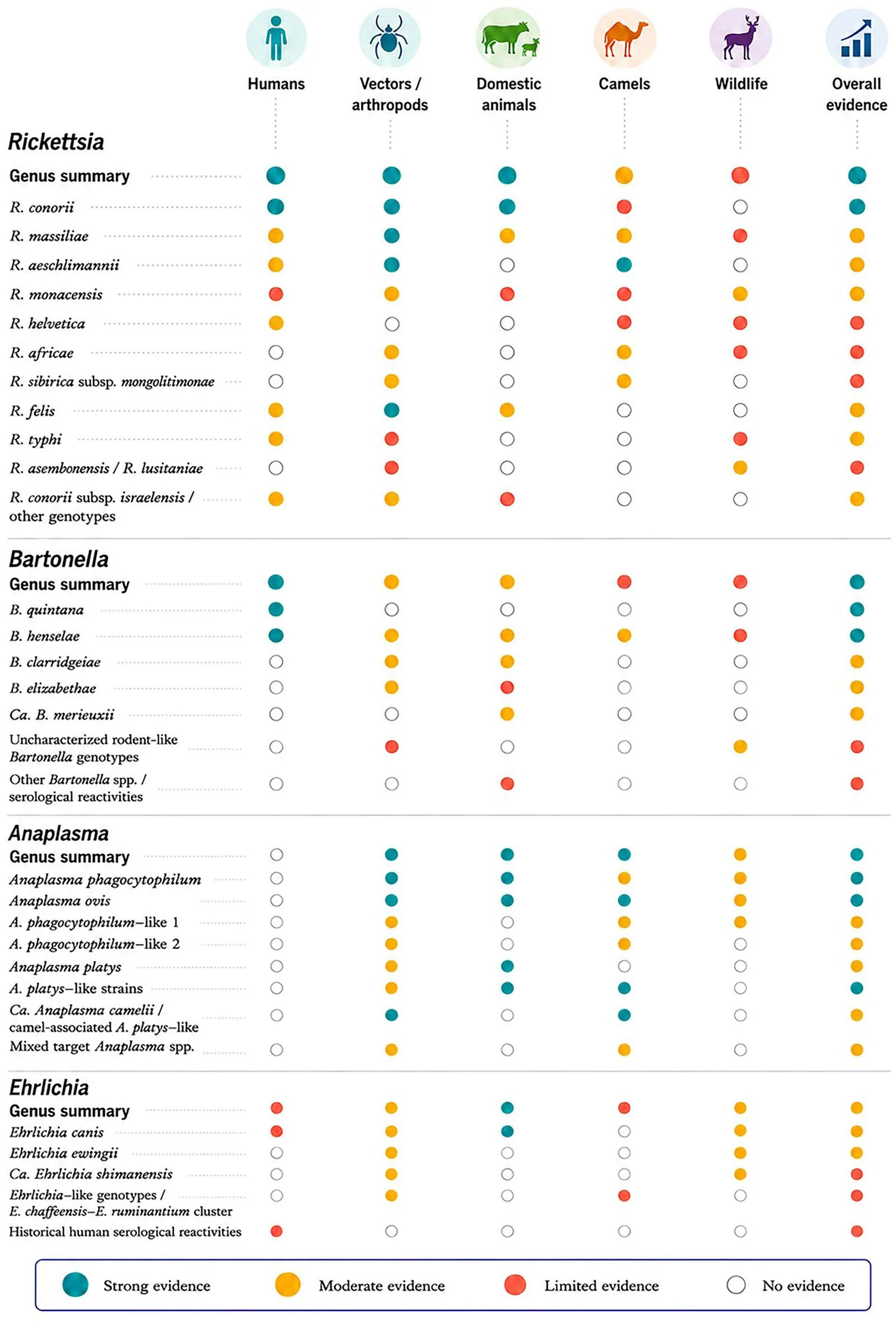
One Health evidence map of *Rickettsia*, *Bartonella*, *Anaplasma* and *Ehrlichia* spp. reported in Tunisia. The figure shows the qualitative strength of Tunisian evidence for the main *Rickettsia*, *Bartonella*, *Anaplasma* and *Ehrlichia* taxa across One Health compartments, including humans, vectors/arthropods, domestic animals, livestock/camels, wildlife and overall evidence. Evidence was classified as strong, moderate, limited or absent/not documented according to the consistency of reports, diagnostic robustness, taxonomic resolution, compartment coverage and clinical or epidemiological relevance. Genus-level summary rows provide an integrated overview of each bacterial group. These evidence levels do not represent prevalence estimates or quantified transmission risk.

**FIGURE 3:**
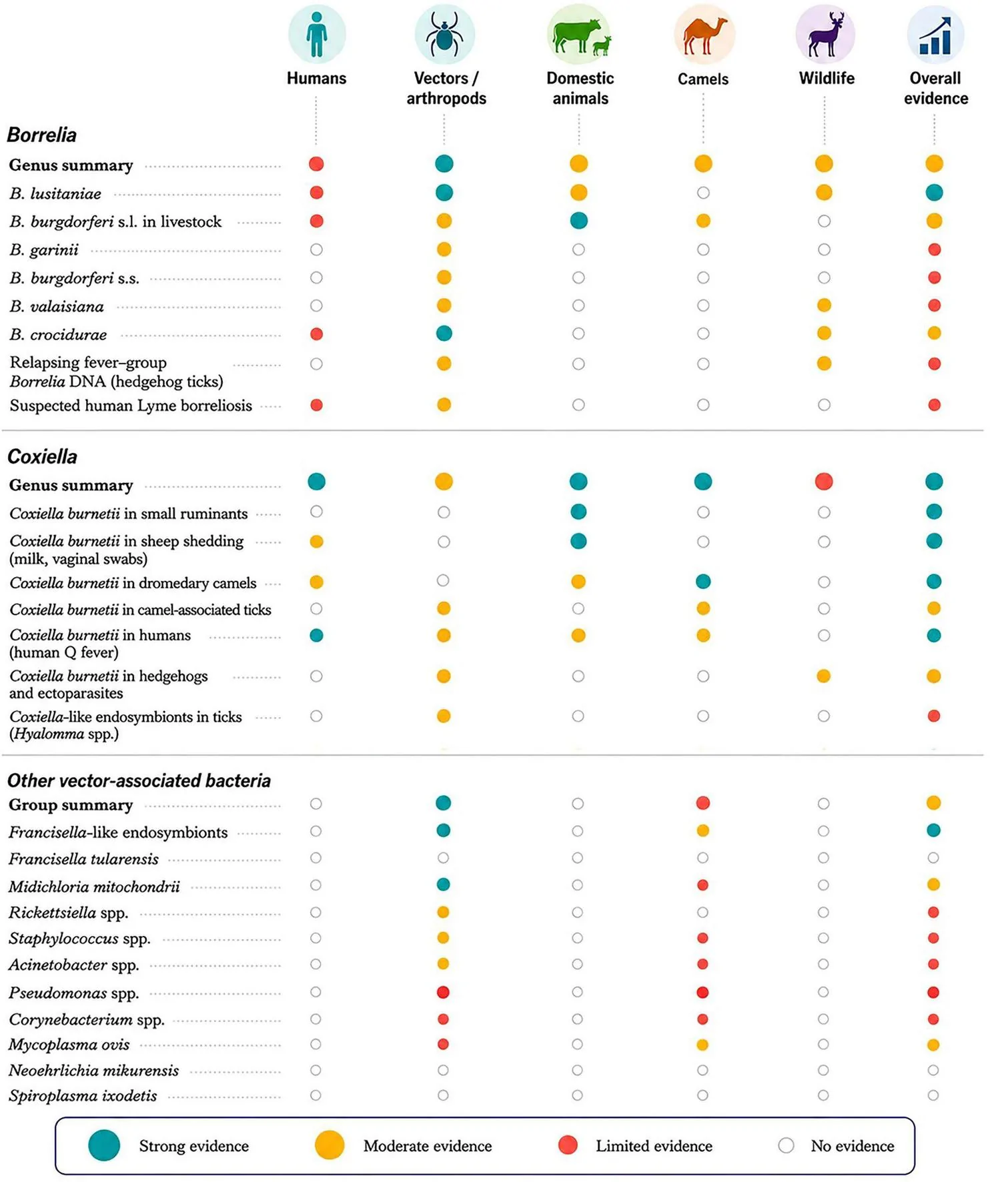
One Health evidence map of *Borrelia*, *Coxiella* and other vector-associated bacteria reported in Tunisia. The figure shows the qualitative strength of Tunisian evidence for *Borrelia* spp., *C. burnetii*/*Coxiella*-like organisms and other vector-associated bacteria across One Health compartments, including humans, vectors/arthropods, domestic animals, livestock/camels, wildlife and overall evidence. Evidence was classified as strong, moderate, limited or absent/not documented according to the consistency of reports, diagnostic robustness, taxonomic resolution, compartment coverage and clinical or epidemiological relevance. Genus- and group-level summary rows highlight the dominant evidence profile of each bacterial group and distinguish confirmed or suspected zoonotic signals from ecological, microbiome or endosymbiotic findings. These evidence levels do not represent prevalence estimates or quantified transmission risk.

**TABLE 1:** One Health evidence matrix for *Rickettsia*, *Bartonella*, *Anaplasma* and *Ehrlichia* spp. reported in Tunisia.

Bacteria genus	Taxon / species / genotype	Main Tunisian evidence	Main One Health compartments	Human evidence in Tunisia	Vector / ectoparasite / environmental evidence	Evidence strength	Interpretation	Key references
*Rickettsia* spp.	*R. conorii*	Historically dominant agent of Mediterranean spotted fever; documented by clinical, serological and molecular studies	Humans, dogs, *Rh. sanguineus* s.l.	Strong human evidence, including MSF and severe manifestations	Mainly dog-associated *R. sanguineus* s.l.	Strong	Core clinically established rickettsial pathogen in Tunisia	Letaief et al., 1995; Znazen et al., 2013; Khrouf et al., 2016; Lamloumi et al., 2024; Gharbi et al., 2024; Taher et al., 2025
	*R. massiliae*	Repeated detection in ticks and reported in human molecular investigations	Humans, dogs, livestock ticks, wildlife-associated ectoparasites	Human molecular evidence reported	Mainly *Rhipicephalus* ticks; also livestock- and wildlife-associated ectoparasites	Moderate to strong	Important non-*conorii* SFG species; true clinical burden remains incompletely defined	Khrouf et al., 2014; Khrouf et al., 2016; Belkahia et al., 2021; Balti et al., 2021; Kratou et al., 2023; Kratou et al., 2025a
	*R. aeschlimannii*	Detected mainly in *Hyalomma* ticks, especially camel- and livestock-associated systems	Dromedaries, cattle, *Hyalomma* ticks	Human serological evidence reported	*Hy. dromedarii* and other *Hyalomma* spp.	Moderate	Relevant for arid-zone and pastoral interfaces; human relevance requires further confirmation	Znazen et al., 2006; Demoncheaux et al., 2012; Selmi et al., 2020b; Kratou et al., 2023
	*R. monacensis*	Detected in *Ixodes* ticks and later in forest, livestock and wildlife-associated contexts	*Ixodes* ticks, rodents, red fox, small-ruminant ticks	No well-characterized clinical burden	*I. ricinus*, *I. inopinatus* and some livestock-associated ticks	Moderate	Marker of northern forest and wildlife interfaces beyond the classical MSF model	Sfar et al., 2008; Belkahia et al., 2021; Selmi et al., 2021a; Kratou et al., 2025b; Selmi et al., 2025
	*R. helvetica*	Detected in *Ixodes* ticks and wildlife-associated contexts	*Ixodes* ticks, rodents, forest-associated wildlife	No clearly established human disease	Mainly *Ixodes* spp.	Limited to moderate	Supports the relevance of northern humid forest ecosystems; clinical significance remains uncertain	Sfar et al., 2008; Selmi et al., 2020b; Selmi et al., 2021a; Kratou et al., 2025a
	*R. felis*	Reported in humans, fleas and recently in bed bugs	Humans, fleas, domestic/peri-domestic arthropods	Human serological or molecular evidence reported	*C. felis*; DNA also reported in *Cimex lectularius*	Moderate	Important flea-associated domestic signal; bed bug detection does not prove transmission	Znazen et al., 2006; Znazen et al., 2013; Khrouf et al., 2014; Selmi et al., 2024a
	*R. typhi*	Historical and clinical evidence of exposure or infection	Humans, possible rodent-flea interface	Human serological and clinical/molecular evidence reported	Classical rodent-flea cycle suspected but insufficiently documented locally	Moderate	Should remain in differential diagnosis of febrile illness; current reservoir and flea data are limited	Letaief et al., 1995; Znazen et al., 2006; Znazen et al., 2013; Khrouf et al., 2016; Selmi et al., 2021a
	Other or sporadic *Rickettsia* taxa	Includes *R. africae*, *R. sibirica* subsp. *mongolitimonae*, *R. asembonensis*, *R. lusitaniae* and other genotypes	Ticks, fleas, wildlife, livestock-associated ectoparasites	No established human burden	Mainly vector- or wildlife-associated detections	Limited	Useful ecological signals, but should not be overinterpreted without clinical linkage	Balti et al., 2021; Selmi et al., 2020b; Selmi et al., 2021a; Kratou et al., 2023; Selmi et al., 2025
*Bartonella* spp.	*B. quintana*	Dominant *Bartonella* species in hospital-based blood culture-negative endocarditis cohorts	Humans; suspected ectoparasite-associated transmission	Strong evidence in blood culture-negative endocarditis	Local vector route not demonstrated	Strong for human disease	Major clinical signal; transmission route remains unresolved	Znazen et al., 2005; Hammami et al., 2013
	*B. henselae*	Reported in human disease and detected in fleas, dromedaries and *Rattus rattus*	Humans, fleas, cats/dogs context, camels, rodents	Human endocarditis and atypical cat-scratch disease evidence reported	Flea-associated DNA; camel and rodent molecular signals	Moderate to strong	Clinically relevant species linking domestic, peri-domestic, camel and rodent interfaces	Znazen et al., 2005; Hammami et al., 2013; El Ouni et al., 2015; Zouari et al., 2017; Selmi et al., 2020a; Selmi et al., 2021a
	*B. clarridgeiae*	Detected mainly in fleas from domestic animals; serological evidence in stray dogs	Fleas, dogs, cats/domestic animal interface	No confirmed human case in the reviewed corpus	*Ctenocephalides* spp. and/or domestic animal flea pools	Moderate	Domestic and peri-domestic zoonotic candidate requiring stronger pet-flea-human linkage	Belkhiria et al., 2017; Zouari et al., 2017
	*B. elizabethae*	Detected in fleas from domestic animals	Fleas, domestic animal environments, possible rodent-related ecology	No local human case documented	Flea-associated DNA detection	Limited to moderate	Flea-associated molecular signal; human relevance remains uncharacterized	Zouari et al., 2017
	*Ca.* B. merieuxii	Molecularly detected in Tunisian stray dogs	Stray dogs	No human evidence	No local vector confirmed	Moderate	Supports stray dogs as sentinels of domestic *Bartonella* diversity	Belkhiria et al., 2017
	Rodent-like or uncharacterized *Bartonella* genotypes	Detected in flea pools or suggested by rodent-associated molecular signals	Fleas, rodents, domestic-wildlife interfaces	No human evidence	Flea-associated and rodent-associated molecular signals	Limited	Indicates hidden *Bartonella* diversity at domestic, peri-domestic and wildlife interfaces	Zouari et al., 2017; Selmi et al., 2021a
	Other serological reactivities	Serological reactivity in stray dogs against several *Bartonella* species	Stray dogs	No direct human evidence linked to these reactivities	Not linked to a confirmed local vector route	Limited / uncertain	Serology may cross-react and should be interpreted cautiously without PCR confirmation	Belkhiria et al., 2017
*Anaplasma* spp.	*A. phagocytophilum*	Molecular and/or serological evidence in horses, cattle, small ruminants, camels and ticks	Horses, cattle, sheep, goats, camels, ticks	No confirmed human cases, PCR detections or serosurveys	*Ixodes*, *Rhipicephalus*, *Hyalomma* and tortoise-associated ticks	Strong animal/tick evidence; absent human evidence	Confirmed zoonotic species globally, but human relevance in Tunisia remains undocumented; broad ruminant surveys also highlight possible confusion with *A. phagocytophilum*-like genotypes.	Sarih et al., 2005; M’ghirbi et al., 2012, 2016, 2022; Ben Said et al., 2014; Rjeibi et al., 2022
	*A. ovis*	Highly prevalent in sheep and goats; detected in camels, rodents, rat fleas and ticks	Small ruminants, camels, ticks, rodents	No Tunisian human evidence	*Rhipicephalus* ticks and one rat flea pool reported	Strong animal molecular evidence	Mainly veterinary pathogen with suspected zoonotic relevance; human role remains unproven	Belkahia et al., 2014; Ben Said et al., 2015; Belkahia et al., 2017; Belkahia et al., 2018; Selmi et al., 2020c; Ben Said et al., 2020; Selmi et al., 2021a; ElHamdi et al., 2022; M’ghirbi et al., 2022
	*A. phagocytophilum*-like genotypes	Detected in small ruminants, *Rhipicephalus* ticks and wildlife-associated contexts	Sheep, goats, ticks, rodents	No human evidence	Mainly *Rhipicephalus* ticks and wildlife-associated detections	Limited to moderate	Related to *A. phagocytophilum*, but zoonotic relevance and pathogenicity remain uncertain	Ben Said et al., 2015; Ben Said et al., 2017a; Belkahia et al., 2017; Belkahia et al., 2019; Selmi et al., 2021a
	*A. platys* / *A. platys*-like / camel-associated *Anaplasma*	Detected in dogs, dromedaries, cattle, sheep and goats; camel strains related to *A. platys*-like organisms	Dogs, dromedaries, ruminants, ticks	No confirmed human cases	*Hyalomma* and *Rhipicephalus* tick systems	Moderate to strong animal molecular evidence	Emerging One Health signal; taxonomy, pathogenicity and zoonotic relevance remain unresolved	Sarih et al., 2005; Belkahia et al., 2015; Ben Said et al., 2017b; Ben Said et al., 2018; Selmi et al., 2019b
	Mixed or contextual *Anaplasma* spp.	Natural infections reported in military or mixed livestock settings	Camels, horses, sheep, ticks and flies	No human evidence	Ticks and flies investigated	Limited / contextual	Useful occupational and veterinary signal, but species-level interpretation may be uncertain	Selmi et al., 2019a
*Ehrlichia* spp.	*E. canis*	Strong historical serological evidence in dogs; old serological signal in febrile humans. Molecular detection in bats	Dogs, *Rh. sanguineus* s.l., domestic/peri-domestic settings	Human seropositivity reported, but no molecular confirmation	Mainly associated with *Rh. sanguineus* s.l.	Strong canine serological evidence; limited human evidence	Main *Ehrlichia* signal in Tunisia, but human disease remains unconfirmed	Ghorbel et al., 1991; Ghorbel et al., 1994; Selmi et al., 2021a
	*E. ewingii*	Molecular detection in North African hedgehogs and/or associated ticks	Hedgehogs, wildlife-associated ticks	No human cases reported	Hedgehog-associated tick system	Moderate molecular evidence	Confirmed zoonotic species globally, but Tunisian evidence remains wildlife-associated	Balti et al., 2021
	*Ca.* E. shimanensis	DNA detected in North African hedgehog tissues	Hedgehogs, wildlife/peri-domestic interface	No human evidence	Vector not clearly identified locally	Limited to moderate	Emerging wildlife-associated taxon with unresolved pathogenicity and vector range	Balti et al., 2021
	*Ehrlichia*-like genotypes	DNA detected in *I. ricinus* and *Hy. scupense*	Ticks, bovine-associated tick context	No human evidence	*I. ricinus*, *Hy. scupense*	Moderate tick-associated molecular evidence	Indicates hidden *Ehrlichia* diversity; species identity and zoonotic meaning remain uncertain	Sarih et al., 2005
	Other or negative *Ehrlichia* evidence	Some livestock and camel-associated tick surveys were negative or weakly informative	Livestock, camels, ticks	No human evidence	Negative or limited tick/host screening	Limited / negative	Helps define gaps, but absence of detection is not definitive absence	Sarih et al., 2005

**TABLE 2:** Critical evidence gaps, surveillance implications and research priorities for *Rickettsia*, *Bartonella*, *Anaplasma* and *Ehrlichia* spp. in Tunisia.

Bacterial group	Critical gap	Why it matters	Surveillance / research implication	Priority	Key references
*Rickettsia* spp.	Human data remain dominated by Mediterranean spotted fever, while the burden of non-*conorii* rickettsioses is poorly characterized	Non-classical rickettsioses may be underdiagnosed or misclassified as classical MSF or non-specific febrile illness	Expand species-level molecular diagnosis in patients with fever, rash, eschar or compatible tick exposure	High	Letaief et al., 1995; Znazen et al., 2013; Khrouf et al., 2016; Lamloumi et al., 2024; Taher et al., 2025
	Most arthropod evidence is based on DNA detection rather than confirmed vector competence	Tick, flea or bed bug positivity may overestimate the role of some arthropods as biological vectors	Conduct longitudinal and experimental studies, especially for unusual or emerging arthropod associations	High	Sfar et al., 2008; Belkahia et al., 2021; Kratou et al., 2023; Selmi et al., 2024b; Kratou et al., 2025a
	Wildlife data remain opportunistic or geographically limited	The role of rodents, hedgehogs and wild carnivores as reservoirs or bridge hosts remains uncertain	Implement systematic wildlife and ectoparasite surveillance across forest, rural and peri-domestic interfaces	Medium / High	Balti et al., 2021; Selmi et al., 2021a; Selmi et al., 2025
	Human, dog, livestock, tick and wildlife data are rarely integrated	Fragmented data limit risk interpretation and early warning	Link clinical diagnosis, dog sentinel surveillance, livestock-associated tick screening and wildlife monitoring within a One Health platform	High	Znazen et al., 2013; Khrouf et al., 2016; Belkahia et al., 2021; Gharbi et al., 2024; Kratou et al., 2025b
*Bartonella* spp.	Human evidence is mainly retrospective and concentrated in hospital-based endocarditis cohorts	The national burden, regional distribution and clinical spectrum of bartonellosis remain poorly defined	Conduct prospective multicenter studies of blood culture-negative endocarditis and compatible febrile or neurological syndromes	High	Znazen et al., 2005; Hammami et al., 2013; El Ouni et al., 2015
	The local transmission route of *B. quintana* is unresolved	Classical louse-borne assumptions may not fully explain rural or animal-exposed patients	Investigate lice, fleas and other ectoparasites in human exposure settings	High	Znazen et al., 2005; Hammami et al., 2013; Zouari et al., 2017
	Fleas carry *Bartonella* DNA, but local vector competence is not experimentally demonstrated	Molecular association may be confused with confirmed transmission	Combine flea screening with longitudinal pet/human studies and experimental or transmission-focused approaches	High	Belkhiria et al., 2017; Zouari et al., 2017
	Owned cats, owned dogs, livestock and camel-exposed populations remain insufficiently studied	Domestic and arid-zone transmission pathways may be underestimated	Expand pet, stray animal, livestock, camel and ectoparasite surveys, especially in high-exposure settings	Medium / High	Belkhiria et al., 2017; Zouari et al., 2017; Selmi et al., 2020a; Selmi et al., 2021a
*Anaplasma* spp.	No confirmed human anaplasmosis cases, PCR detections or human serosurveys were documented in the reviewed Tunisian corpus	Strong animal and tick evidence cannot be translated into public health burden	Screen febrile patients with tick exposure and high-risk occupational groups using serology and molecular tools	High	M’ghirbi et al., 2012; Ben Said et al., 2014; Ben Said et al., 2018; M’ghirbi et al., 2022; Rjeibi et al., 2022
	The zoonotic relevance of *A. ovis* remains suspected but unproven	Overstatement may create misleading public health conclusions	Use cautious terminology and prioritize targeted human surveys in exposed livestock and camel-contact populations	High	Belkahia et al., 2014; Belkahia et al., 2017; Selmi et al., 2020c; Selmi et al., 2021a; ElHamdi et al., 2022; M’ghirbi et al., 2022
	*A. phagocytophilum*-like, *A. platys*-like, camel-associated strains and genetically diverse *A. ovis* isolates remain incompletely resolved	Misclassification may exaggerate or obscure zoonotic risk and may complicate comparison between animal, tick and human data	Apply discriminatory molecular tools, multilocus typing and, where possible, whole-genome sequencing	Medium / High	Belkahia et al., 2015; Ben Said et al., 2015; Ben Said et al., 2017a; Ben Said et al., 2020; Belkahia et al., 2019; Selmi et al., 2019a
	Several tick species carry *Anaplasma* DNA, but vector competence is not demonstrated	Arthropod DNA detection may be mistaken for proven biological transmission	Conduct longitudinal and experimental vector competence studies on *Ixodes*, *Rhipicephalus* and *Hyalomma* ticks	High	Sarih et al., 2005; Belkahia et al., 2018; Selmi et al., 2019b; Rjeibi et al., 2022
*Ehrlichia* spp.	No molecularly confirmed human ehrlichiosis case was documented in the reviewed Tunisian corpus	The real human burden remains unknown despite old serological suspicion	Include *Ehrlichia* spp. in molecular panels for febrile patients with tick exposure	High	Ghorbel et al., 1991; Ghorbel et al., 1994; Sarih et al., 2005
	Human and canine serology may cross-react among related *Ehrlichia* species	Serology alone cannot reliably identify the infecting species	Combine paired serology with PCR, sequencing and species-specific molecular markers	High	Ghorbel et al., 1991; Ghorbel et al., 1994
	Canine *E. canis* strains are insufficiently characterized molecularly	The zoonotic relevance and strain diversity of the dog-tick cycle remain unclear	Compare dog, tick and suspected human strains using multilocus markers	Medium / High	Ghorbel et al., 1994
	Wildlife-associated detections of *E. ewingii* and *Ca.* E. shimanensis are recent and geographically limited	Emerging wildlife signals may be overinterpreted or missed clinically	Expand seasonal wildlife and ectoparasite sampling and compare strains across wildlife, dogs, ticks and humans	Medium / High	Balti et al., 2021

**TABLE 3:** One Health evidence matrix for *Borrelia* spp., *Coxiella* spp. and other vector-associated bacteria reported in Tunisia.

Bacterial group	Taxon / species / genotype	Main Tunisian evidence	Main One Health compartments	Human evidence in Tunisia	Vector / ectoparasite / environmental evidence	Evidence strength	Interpretation	Key references
*Borrelia* spp.	*B. lusitaniae*	Dominant Lyme-group genospecies detected in Tunisian *Ixodes* ticks	*I. ricinus*, *I. inopinatus*, forest ecosystems	Human pathogenicity in sufficiently confirmed	Northern humid/sub-humid *Ixodes* systems	Strong vector/environmental evidence; limited human confirmation	Key Lyme-group agent for northern surveillance; clinical relevance needs human molecular evidence	Younsi et al., 2001; Younsi et al., 2005; Selmi et al., 2024a
	*B. burgdorferi* s.l. complex in livestock	Molecular detection in goats, sheep, cattle and camels	Livestock, camels, livestock-associated ticks	Human evidence mainly serological or clinically suspected	Livestock-associated molecular signal	Moderate animal molecular evidence	Suggests exposure or circulation, but reservoir competence remains unproven	Ben Said et al., 2016
	Other Lyme-group genospecies	Rare or limited reports of *B. garinii*, *B. burgdorferi* s.s. and *B. valaisiana*	Mainly *Ixodes* ticks in northern ecosystems	No direct human molecular evidence	*Ixodes* tick-associated detections	Limited	Broader Lyme-group diversity exists, but these genospecies remain minor compared with *B. lusitaniae*	Younsi et al., 2005
	*Borrelia crocidurae*	Molecularly detected in *O. erraticus* from rodent burrows	Soft ticks, rodent-burrow environments	Recent human relapsing fever cases poorly documented or possibly underdiagnosed	*O. erraticus*; *O. normandi* negative in available study	Moderate to strong vector evidence	Key relapsing fever-group signal for southern rural and Saharan surveillance	Bouattour et al., 2010
	Relapsing fever-group *Borrelia* DNA	Detected in ticks collected from North African hedgehogs; hedgehog biopsies negative	Hedgehog-associated ticks, wildlife/peri-domestic interface	No direct human evidence	Wildlife-associated tick DNA detection	Limited	Suggests wildlife-tick interface, but not hedgehog reservoir competence	Balti et al., 2021
	Suspected human Lyme borreliosis	Clinical case reports and pediatric cases based mainly on serology	Humans, suspected tick exposure	Serological or clinical evidence reported; limited direct molecular confirmation	Vector exposure suspected but not always confirmed	Limited clinical evidence	Supports clinical awareness, but national burden and genospecies attribution remain uncertain	Mahfoudhi et al., 2015
*Coxiella* spp.	*C. burnetii* in humans	Human Q fever documented, including pneumonia, hepatitis and chronic forms	Humans, livestock-exposed populations	Strong clinical evidence, mainly hospital-based	Environmental / livestock exposure more relevant than classical vector transmission	Strong for clinical existence; limited for national incidence	Major One Health pathogen, probably underdiagnosed and insufficiently integrated into surveillance	Ben Arab et al., 2008; Kaabia and Letaief, 2009; Kaabia and Bahloul, 2021
	*C. burnetii* in small ruminants	Serological and molecular evidence in sheep and goats, including abortion contexts, milk and vaginal swabs	Sheep, goats, reproductive samples, milk, farm environments	Indirect relevance through occupational/ environmental exposure	Birth products, milk and farm contamination	Strong animal and shedding evidence	Central livestock compartment for Q fever risk, especially abortion and parturition	Rekiki et al., 2005; Barkallah et al., 2018; Guesmi et al., 2023
	*C. burnetii* in dromedary camels	High serological exposure reported in camels; camel-associated ticks investigated	Dromedaries, camel systems, *Hyalomma* ticks	Indirect relevance for camel-exposed populations	Camel-associated *Hyalomma* ticks and potential raw milk/parturition exposure	Strong serological evidence; moderate transmission evidence	Important arid-zone compartment, but shedding routes and camel-to-human transmission remain unresolved	Selmi et al., 2018; Selmi et al., 2019c
	Tick-, wildlife- and ectoparasite-associated *C. burnetii* detections	DNA detected in camel-associated *Hyalomma* ticks and in hedgehogs/ectoparasites	Ticks, fleas, hedgehogs, peri-domestic wildlife	No direct human cases linked to these compartments	*Hyalomma* ticks, hedgehog-associated ticks/fleas	Moderate molecular evidence	Useful ecological signal, but not proof of tick-borne Q fever transmission	Selmi et al., 2019a; Balti et al., 2021
	*Coxiella*-like organisms / endosymbionts	*Coxiella*-like endosymbionts not detected in tested cattle-associated *Hyalomma* ticks	Tick microbiome	Not applicable	Negative or microbiome-based evidence	Limited / negative	Helps avoid confusion between pathogenic *C. burnetii* and tick endosymbionts	Benyedem et al., 2022
Other vector-associated bacteria	*Francisella*-like endosymbionts	Dominant bacterial signals in *Hyalomma* tick microbiomes	*Hyalomma* ticks, tick microbiome	No human clinical evidence	Tick microbiome/ endosymbiont signal	Strong endosymbiont evidence	Important tick microbiome component; should not be confused with pathogenic *F. tularensis*	Benyedem et al., 2022; Kratou et al., 2025a
	*F. tularensis*	Not specifically isolated or molecularly confirmed in the reviewed Tunisian corpus	Not demonstrated locally	No human evidence	No confirmed Tunisian vector or host detection in this corpus	No direct Tunisian evidence	Relevant only as a differential concern; genus-level *Francisella* reads should not be interpreted as tularemia	Benyedem et al., 2022; Kratou et al., 2025b
	*M. mitochondrii*	Detected in Tunisian *Ixodes* and *Hyalomma* ticks; frequent in adult *Ixodes* from northwestern Tunisia	*Ixodes* ticks, *Hyalomma* ticks, tick microbiome	No human or animal disease evidence	Tick endosymbiont signal	Strong endosymbiont evidence	Frequent tick endosymbiont of ecological interest; epidemiological impact remains unproven	Benyedem et al., 2022; Jlassi et al., 2026
	*Rickettsiella* spp.	Molecular detection in questing *I. ricinus* and *I. inopinatus*	*Ixodes* ticks, forest tick microbiome	No human or animal infection evidence	Questing tick molecular detection	Moderate tick-associated molecular evidence	Emerging tick-associated signal; zoonotic relevance remains unknown	Jlassi et al., 2026
	Opportunistic bacterial genera	*Staphylococcus*, *Acinetobacter*, *Pseudomonas*, *Corynebacterium* and related genera detected in tick microbiomes	Tick microbiome, cattle- and camel-associated ticks	No human evidence linked to tick exposure	16S rRNA microbiome signals	Limited to moderate microbiome evidence	May represent environmental, host-skin or transient signals; species-level pathogenicity and transmission are unproven	Benyedem et al., 2022; Kratou et al., 2025a
	*Neoehrlichia mikurensis*	Specifically screened but not detected in northwestern Tunisian *Ixodes* ticks	*I. ricinus*, *I. inopinatus*	No human evidence	Negative *Ixodes* screening	Informative negative evidence	Useful baseline, but absence cannot be generalized nationally	Jlassi et al., 2026
	*Spiroplasma ixodetis*	Specifically screened but not detected in northwestern Tunisian *Ixodes* ticks	*I. ricinus*, *I. inopinatus*	No human evidence	Negative *Ixodes* screening	Informative negative evidence	Low current priority, but useful baseline for tick microbiome surveillance	Jlassi et al., 2026
	*Mycoplasma ovis*	First molecular detection and genetic characterization in Tunisian small ruminants; detected only in sheep, with marked regional variation and highest prevalence in central Tunisia	Sheep, small ruminant systems, livestock-associated arthropod exposure	No human evidence in Tunisia	Blood-feeding arthropod transmission is biologically suspected; ticks were collected from sheep, but no significant association between tick infestation and *M. ovis* prevalence was demonstrated	Moderate livestock molecular evidence; limited vector evidence	Relevant hemotropic bacterial signal in small ruminants; should be treated as an arthropod-associated veterinary pathogen with uncertain zoonotic significance rather than as an absent or undocumented taxon	Rjeibi et al., 2015

**TABLE 4:** Critical evidence gaps, surveillance implications and research priorities for *Borrelia* spp., *Coxiella burnetii* and other vector-associated bacteria in Tunisia.

Bacterial group	Critical gap	Why it matters	Surveillance / research implication	Priority	Key references
*Borrelia* spp.	Human Lyme borreliosis evidence remains mainly serological or clinically suspected	The true burden and genospecies attribution of human Lyme borreliosis remain uncertain	Introduce PCR and sequencing for selected suspected human cases, especially from CSF, skin or synovial samples when available	High	Mahfoudhi et al., 2015
	*B. lusitaniae* dominates Tunisian *Ixodes* ticks, but its local pathogenicity is insufficiently confirmed	Environmental circulation may be overinterpreted as direct human clinical risk	Link human cases to genospecies through molecular confirmation and strain comparison	High	Younsi et al., 2001; Younsi et al., 2005; Selmi et al., 2024a
	*Ixodes inopinatus* carries *B. lusitaniae* DNA, but vector competence is unknown	Risk assessment may change if this species is confirmed as a biological vector	Conduct vector competence studies and longitudinal monitoring of *Ixodes* populations	Medium / High	Selmi et al., 2024b
	*B. crocidurae* circulates in *O. erraticus*, but recent human relapsing fever is poorly documented	Tick-borne relapsing fever may be underdiagnosed in rural or Saharan settings	Improve clinical awareness and molecular diagnosis for recurrent fever in southern and arid regions	High	Bouattour et al., 2010; Balti et al., 2021
*Coxiella burnetii*	Human Q fever is documented but probably underestimated	Non-specific symptoms and lack of systematic notification obscure national incidence	Include Q fever in differential diagnosis of fever, pneumonia, hepatitis and culture-negative endocarditis	High	Ben Arab et al., 2008
	Livestock studies often focus on abortion-history flocks or selected farms	Prevalence may not represent the general animal population	Conduct population-based surveys in healthy and symptomatic herds across ecological zones	Medium / High	Rekiki et al., 2005; Barkallah et al., 2018; Guesmi et al., 2023
	Serology alone cannot attribute abortion or shedding to active infection	Veterinary and public health impact may be misestimated	Use PCR on placentas, aborted fetuses, vaginal swabs, milk and environmental samples	High	Rekiki et al., 2005; Barkallah et al., 2018; Guesmi et al., 2023
	Tick-associated detections may lead to misclassification as a classical tick-borne pathogen	The major route is likely environmental/aerosol rather than vector-borne	Use cautious terminology and focus surveillance on livestock, reproductive materials, milk, dust and exposed humans	High	Selmi et al., 2019a; Balti et al., 2021; Kaabia and Bahloul, 2021
Other vector-associated bacteria	No human clinical cases, serological evidence or molecular confirmation were reported for these taxa in Tunisia	Zoonotic relevance remains speculative despite tick microbiome detections	Conduct targeted screening only where ecological or occupational exposure justifies it	Medium	Benyedem et al., 2022; Jlassi et al., 2026; Kratou et al., 2025b
	16S rRNA microbiome data often identify bacteria only at genus level	Harmless endosymbionts may be confused with pathogenic species, especially *Francisella*-like organisms versus *F. tularensis*	Use species-specific PCR, multilocus sequencing or whole-genome sequencing	High	Benyedem et al., 2022; Kratou et al., 2025a
	Dominant *Francisella* reads probably represent endosymbionts, but pathogenic *F. tularensis* must be excluded	Overinterpretation may falsely suggest tularemia circulation	Develop a targeted workflow distinguishing *Francisella*-like endosymbionts from pathogenic *F. tularensis*	High	Benyedem et al., 2022; Kratou et al., 2025a
	Opportunistic genera detected in tick microbiomes lack species-level and functional confirmation	These bacteria may be environmental or skin-associated rather than vector-borne pathogens	Combine culture, species-level identification, anti microbial resistance profiling and host-source comparison	Medium	Benyedem et al., 2022; Kratou et al., 2025b
	The epidemiology, clinical impact and arthropod transmission of *M. ovis* in Tunisian small ruminants remain poorly characterized	The only available molecular study detected *M. ovis* in sheep, especially in central Tunisia, but goats were negative and the role of ticks or other blood-feeding arthropods was not clarified	Expand molecular screening across sheep and goat populations, include hematological and clinical data, and investigate potential mechanical or biological arthropod vectors, including ticks and biting flies	Medium	Rjeibi et al., 2015
Cross-cutting	Human, veterinary, entomological, wildlife, environmental and microbiome data remain poorly connected	Fragmented evidence prevents robust risk interpretation and public health prioritization	Establish a national One Health evidence platform linking clinical cases, animal sentinels, arthropods, wildlife, environment and sequence data	High	All studies reviewed in Tables 1, 3
	Detection is frequently easier to document than transmission	Molecular positivity may be confused with infection, reservoir competence, vector competence or human disease	Apply a hierarchy of evidence distinguishing exposure, molecular detection, active infection, competence and confirmed disease	High	Sarih et al., 2005; Bouattour et al., 2010; Belkahia et al., 2019; Selmi et al., 2019b; Benyedem et al., 2022; Selmi et al., 2024a
	Geographical and ecological coverage is uneven	National conclusions may be biased toward well-studied northern, coastal, livestock or camel-associated settings	Expand surveillance to under-sampled central, southern, Saharan, peri-urban and wildlife interfaces	Medium / High	Demoncheaux et al., 2012; Belkahia et al., 2021; Selmi et al., 2020a,2021b,2020c; Selmi et al., 2021a; Kratou et al., 2025a
	Negative findings are often underused	Absence of detection in one setting is informative but not equivalent to national absence	Report negative findings with clear sampling context, sample size, methods and ecological limits	Medium	Bouattour et al., 2010; Selmi et al., 2020a,2021b,2020c; Jlassi et al., 2026; Benyedem et al., 2022

### 3.1 *Rickettsia* spp.

Among vector-borne bacterial pathogens reported in Tunisia, *Rickettsia* spp. represent one of the most structured and clinically relevant evidence bases. Historically, Tunisian rickettsiology was dominated by Mediterranean spotted fever (MSF), a disease primarily associated with *Rickettsia conorii*. The classical transmission cycle involves the brown dog tick *Rhipicephalus sanguineus* sensu lato, which has historically been considered the main vector in the Mediterranean region (Letaief et al., 1995; Znazen et al., 2013; Lamloumi et al., 2024). However, the progressive use of serology, molecular diagnosis, sequencing and multispacer typing has expanded this classical view toward a broader spectrum of *Rickettsia* species detected in humans, domestic animals, livestock, wildlife, and arthropods (Znazen et al., 2006; Znazen et al., 2013; Khrouf et al., 2014; Khrouf et al., 2016; Belkahia et al., 2021; Balti et al., 2021; Kratou et al., 2023, 2025a).

The most established human rickettsial disease in Tunisia remains MSF. Recent hospital-based data confirm that suspected MSF continues to be diagnosed in Tunisian patients, particularly in clinical cases combining fever, rash and inoculation eschar (Lamloumi et al., 2024). *R. conorii* therefore remains the species of greatest clinical and public health importance. Severe presentations should also be considered, as illustrated by the report of Guillain-Barré syndrome associated with *R. conorii* infection, indicating that rickettsioses should not always be regarded as benign febrile illnesses in endemic areas (Taher et al., 2025). At the same time, Tunisian data indicate that human rickettsioses are not limited to *R. conorii*. Serological and molecular investigations have reported evidence compatible with infections caused by or related to *R. massiliae*, *R. helvetica*, *R. monacensis*, *R. aeschlimannii*, *R. felis*, and *R. typhi* (Znazen et al., 2006, 2013; Sfar et al., 2008; Khrouf et al., 2016). However, these species should not be considered epidemiologically equivalent, as they belong to distinct ecological systems (Znazen et al., 2006, 2013; Khrouf et al., 2016; Belkahia et al., 2021). While *R. conorii* is primarily associated with Mediterranean spotted fever and the dog-associated tick *Rh. sanguineus* s.l. (Letaief et al., 1995; Znazen et al., 2013; Lamloumi et al., 2024), *R. massiliae* is mainly linked to *Rhipicephalus* ticks (Khrouf et al., 2014; Khrouf et al., 2016; Belkahia et al., 2021), *R. aeschlimannii* to *Hyalomma* ticks, particularly in camel and livestock-associated environments (Demoncheaux et al., 2012; Selmi et al., 2020b; Kratou et al., 2023), and *R. helvetica* and *R. monacensis* to *Ixodes* ticks (Sfar et al., 2008; Belkahia et al., 2021; Kratou et al., 2025a). In addition, *R. felis* is mainly associated with flea-borne cycles involving *Ctenocephalides felis* (Znazen et al., 2006; Khrouf et al., 2014; Zouari et al., 2017), whereas *R. typhi* belongs to flea-borne murine typhus cycles (Letaief et al., 1995). Therefore, the detection of different rickettsial species in Tunisia reflects multiple ecological niches rather than a single homogeneous transmission cycle. Historical seroepidemiological data also showed exposure to *R. conorii* and *R. typhi* among blood donors in central Tunisia, supporting the long-standing endemicity of both MSF and murine typhus in the country (Letaief et al., 1995). Nevertheless, the true clinical burden of non-*conorii* rickettsioses remains insufficiently defined, mainly because routine diagnosis often remains oriented toward classical MSF and because species-level confirmation is not systematically performed.

Ticks are the best-documented arthropods associated with *Rickettsia* spp. in Tunisia. *Rh. sanguineus* s.l. remains the key tick species in the domestic and peri-domestic cycle of *R. conorii*, particularly through its close association with dogs and human dwellings (Znazen et al., 2013; Gharbi et al., 2024). This tick complex has also been associated with *R. massiliae* and other spotted fever group rickettsiae, supporting its importance beyond the classical MSF framework (Khrouf et al., 2014; Khrouf et al., 2016). Other *Rhipicephalus* species, including *Rh. turanicus*, have been found carrying rickettsial DNA in ticks collected from livestock and forest-associated environments, suggesting that rural and pastoral systems may contribute to the circulation of several spotted fever group rickettsiae (Belkahia et al., 2021; Kratou et al., 2025a).

The genus *Hyalomma* is particularly relevant in arid and livestock-associated settings. Among Tunisian *Hyalomma* species, *Hy. marginatum* is of particular epidemiological importance because it is recognized as a main vector of Crimean-Congo hemorrhagic fever virus (CCHFV) in the Mediterranean region (Wasfi et al., 2016). Adults of *H. marginatum* are relatively large ticks and exhibit an active host-seeking strategy, using environmental cues to locate mammalian hosts (Estrada-Peña et al., 2017; Apanaskevich and Horak, 2008). *R. aeschlimannii* was detected in *Hy. dromedarii* collected from dromedaries in southern Tunisia, while broader screening of cattle- and camel-associated *Hyalomma* ticks revealed additional spotted fever group rickettsiae, including *R. massiliae* and *R. sibirica* subsp. *mongolitimonae* (Demoncheaux et al., 2012; Selmi et al., 2020b; Kratou et al., 2023). These findings are important for pastoral, livestock-rearing areas, but they should be interpreted cautiously. The detection of bacterial DNA in livestock may indicate exposure to infected arthropod vectors, but does not by itself demonstrate that livestock serve as competent reservoirs for human infection (Demoncheaux et al., 2012; Selmi et al., 2020b; Kratou et al., 2023).

In northern humid and forested regions, *Ixodes ricinus* and *Ixodes inopinatus* contribute to a distinct ecological component of the Tunisian rickettsial landscape. The detection of *R. monacensis* and *R. helvetica* in *Ixodes* ticks highlights forest-associated transmission systems that differ from the classical dog-*Rhipicephalus*-Mediterranean spotted fever model (Sfar et al., 2008; Kratou et al., 2025a). Although these findings expand the diversity of known *Rickettsia* species in Tunisia, their local clinical relevance remains insufficiently characterized. Domestic arthropods also contribute to the diversity of rickettsial circulation. *C. felis* has been associated with *R. felis* and other rickettsial DNA detections, supporting consideration of flea-associated cycles in domestic environments involving cats, dogs and humans (Znazen et al., 2006, 2013; Khrouf et al., 2014). More recently, *R. felis* DNA was detected in *Cimex lectularius*, suggesting a possible association with domestic arthropods; however, this observation requires cautious interpretation because molecular detection alone does not demonstrate biological vector competence or human transmission (Selmi et al., 2024b).

Domestic animals, particularly dogs, represent valuable sentinel compartments for assessing exposure to rickettsial agents. The close relationship between dogs and humans, together with their association with *Rh. sanguineus* s.l., supports their relevance for monitoring domestic and peri-domestic transmission contexts. High canine seroprevalence against *R. conorii* in northern Tunisia supports their use as indicators of exposure risk (Gharbi et al., 2024). However, seropositivity reflects previous exposure rather than necessarily active infection, and molecular confirmation in animal blood remains limited. Similarly, livestock-associated tick studies have reported *R. massiliae*, *R. aeschlimannii*, *R. sibirica* subsp. *mongolitimonae* and *R. monacensis* in ticks collected from cattle, sheep and goats (Belkahia et al., 2021; Kratou et al., 2023).

Wildlife data have expanded the ecological interpretation of rickettsial circulation in Tunisia. Wild rodents were found to harbor DNA of several vector-borne bacteria, including rickettsial taxa, suggesting that rodent-associated systems may participate in sylvatic or peri-domestic cycles (Selmi et al., 2021a). The North African hedgehog, Atelerix algirus, has also been investigated as a host for infected ticks and fleas, with detection of rickettsial taxa among other vector-borne agents (Balti et al., 2021). More recently, R. monacensis was reported in a red fox, extending the wildlife signal to wild carnivores and reinforcing the need to consider forest and peri-domestic interfaces (Selmi et al., 2025). These observations are valuable, but the role of rodents, hedgehogs and red foxes as confirmed reservoirs remains insufficiently demonstrated because several studies rely on limited sample sizes, opportunistic sampling or restricted geographical coverage (Balti et al., 2021; Selmi et al., 2021a; Selmi et al., 2025).

The Tunisian evidence on *Rickettsia* spp. also reflects clear diagnostic progress. Earlier studies relied mainly on serology, including immunofluorescence assay and Western blot, while later work incorporated qPCR, reverse line blot, sequencing and multispacer typing (Znazen et al., 2006, 2013; Khrouf et al., 2016). These molecular tools have enabled more precise identification of species and genotypes, moving Tunisian rickettsiology beyond a single-pathogen MSF framework. The use of skin swabs has also been proposed as a useful non-invasive tool for early molecular diagnosis of rickettsial infections, especially when eschar or skin lesions are available (Khrouf et al., 2016). However, diagnostic heterogeneity remains a limitation. Differences between serological, molecular and typing methods complicate comparisons across studies and highlight the need for standardized laboratory protocols and reporting criteria.

Despite being one of the best-documented bacterial groups in Tunisia, *Rickettsia* spp. still present important knowledge gaps that limit the interpretation of their epidemiological significance. A major limitation is that much of the available vector evidence relies on molecular detection of *Rickettsia* DNA in ticks, fleas or other arthropods. Although these findings are essential for identifying potential transmission interfaces, they do not by themselves demonstrate biological vector competence, efficient transmission or direct risk for human infection (Belkahia et al., 2021; Kratou et al., 2023; Selmi et al., 2024b). In addition, available data remain geographically heterogeneous, with studies concentrated mainly in northern forested areas, selected cattle farms, small-ruminant systems, southern camel-associated settings and a limited number of clinical centers, while several governorates and ecological compartments remain insufficiently investigated (Demoncheaux et al., 2012; Belkahia et al., 2021; Selmi et al., 2020b; Kratou et al., 2025a). Wildlife-associated evidence is particularly limited and remains exploratory; systematic investigations of rodents, hedgehogs, wild carnivores and their associated ectoparasites are required before their potential epidemiological roles can be accurately defined (Balti et al., 2021; Selmi et al., 2021a; Selmi et al., 2025). Therefore, although the evidence base for *Rickettsia* spp. is relatively strong compared with several other bacterial groups, particularly for *R. conorii*, it remains uneven across species, regions and One Health compartments. Future surveillance should prioritize the diagnostic approaches, sentinel monitoring, livestock- and associated tick surveillance, vector competence studies and wildlife investigations within a coordinated One Health framework. The taxon-level evidence for *Rickettsia* spp. is summarized in Table 1, while the major knowledge gaps and surveillance priorities are presented in Table 2.

### 3.2 *Bartonella* spp.

In Tunisia, the *Bartonella* evidence base links severe human disease, particularly blood culture-negative endocarditis, with domestic and peri-domestic ectoparasites, stray dogs, cats, dromedary camels and synanthropic rodents (Znazen et al., 2005; Hammami et al., 2013; Belkhiria et al., 2017; Zouari et al., 2017; Selmi et al., 2020a; Selmi et al., 2021a). The available data suggest that bartonellosis should not be viewed as a sporadic clinical curiosity, but as a neglected zoonotic bacterial disease requiring coordinated medical, veterinary and entomological attention.

Hospital-based cohorts from Sfax identified *Bartonella* spp. as etiological agents in approximately 8.9–9.8% of infective endocarditis cases, with *B. quintana* being the dominant species and *B. henselae* reported less frequently (Znazen et al., 2005; Hammami et al., 2013). *B. henselae* is also the main causative agent of cat-scratch disease, typically characterized by a local inoculation skin lesion followed by regional lymphadenopathy (Lin and Saccoccio, 2023), whereas *B. quintana*, mainly transmitted by body lice (*Pediculus humanus corporis*), is an important cause of blood culture-negative endocarditis and trench fever (Puspitasari et al., 2025). These observations highlight the clinical relevance of *Bartonella* infections in Tunisia, particularly in relation to endocarditis. Tunisian patients with *Bartonella* endocarditis showed severe valvular damage and substantial mortality, emphasizing the importance of early diagnosis and appropriate management (Hammami et al., 2013; Letaief et al., 2007; Znazen et al., 2005). In addition, a case of cat-scratch disease associated with Guillain-Barré syndrome suggests that *B. henselae* may be involved in atypical neurological presentations, although isolated case reports should not be interpreted as indicators of population-level frequency (El Ouni et al., 2015).

Beyond human disease, molecular screening of fleas collected from domestic animals revealed zoonotic *Bartonella* species, including *B. henselae*, *B. clarridgeiae* and *B. elizabethae*, in *C. felis*, *Ctenocephalides canis* and *P. irritans* (Zouari et al., 2017). These findings support the importance of domestic and peri-domestic flea-associated systems involving cats, dogs, livestock and humans in Tunisian *Bartonella* ecology. However, the available evidence should be interpreted cautiously, as detection of *Bartonella* DNA in fleas does not demonstrate local biological vector competence or confirm transmission to humans or animals (Zouari et al., 2017). Similarly, although human evidence is relatively strong for *Bartonella*-associated endocarditis, it remains geographically and clinically restricted, with most available data originating from retrospective hospital-based studies in the Sfax region (Znazen et al., 2005; Hammami et al., 2013). Therefore, further integrated investigations are required to better define the epidemiological significance of *Bartonella* circulation in Tunisia.

Available evidence in Tunisia suggests that the role of ticks in *Bartonella* circulation remains unclear. Molecular screening of *Hyalomma* ticks collected from dromedary camels and rodent-associated ectoparasites did not detect *Bartonella* DNA in the investigated samples (Selmi et al., 2020a; Selmi et al., 2021a). However, these negative findings should be interpreted within the specific ecological and methodological context of each study and do not exclude the possibility of tick-associated *Bartonella* circulation under other conditions.

Stray dogs showed substantial serological evidence of exposure to several *Bartonella* species. Molecular detection of *Candidatus* Bartonella merieuxii in Tunisian dogs, a species initially described in Iraq, suggests that dogs may represent potential sentinels of domestic and peri-domestic *Bartonella* diversity (Belkhiria et al., 2017). However, their role as true reservoirs remains unconfirmed, as available cross-sectional PCR-based studies do not demonstrate long-term bacterial maintenance, transmission competence, or zoonotic spillover. Cats are classically recognized as important reservoirs for *B. henselae* and *B. clarridgeiae*, but the Tunisian corpus is more strongly supported by flea-based data than by large-scale molecular surveys of cats themselves (Zouari et al., 2017). Cats should therefore remain part of the One Health interpretation, particularly because cat-associated fleas may carry zoonotic *Bartonella* DNA, but more direct studies on owned and stray cats are still needed.

Furthermore, fleas collected from sheep and goats were found carrying zoonotic *Bartonella* DNA, suggesting that pastoral systems may contribute to exposure through flea-infested animals (Zouari et al., 2017). However, direct molecular surveys of livestock blood or tissues remain limited. Livestock should therefore be discussed mainly as hosts for infected ectoparasites rather than as confirmed mammalian reservoirs. This distinction is important because animal-associated ectoparasite positivity may identify a risk interface without proving that the animal host maintains the bacterium.

A particularly original contribution of Tunisian research is the molecular detection of *B. henselae* DNA in dromedary camels (Selmi et al., 2020a). This finding expands the currently recognized host range of *Bartonella* in Tunisia beyond the classical cat-flea-human interface and suggests that camel-associated ecosystems may represent previously underexplored ecological compartments. From a One Health perspective, the relevance of this finding lies in the position of dromedaries at the interface between animal production systems, arthropod communities and human activities in southern Tunisia, including camel husbandry, transport, slaughtering practices and tourism-related interactions. However, the epidemiological significance of camel-associated *Bartonella* remains unresolved. The detection of *B. henselae* DNA in dromedaries does not demonstrate that camels act as competent maintenance reservoirs or that they contribute directly to human infection. Moreover, *Bartonella* DNA was not detected in the examined *Hyalomma* ticks, leaving the transmission pathway uncertain and suggesting that other arthropod groups, particularly fleas that were not investigated in this study, may contribute to bacterial maintenance and transmission dynamics (Selmi et al., 2020a).

Wildlife evidence for *Bartonella* in Tunisia is more limited than the evidence from humans, fleas and dogs, but it remains relevant for understanding peri-domestic transmission interfaces. *B. henselae* DNA was detected in *Rattus rattus*, suggesting that synanthropic rodents may participate in local *Bartonella* ecology (Selmi et al., 2021a). This observation is important because *R. rattus* often lives near human dwellings, livestock facilities and food-storage environments, potentially linking wildlife, domestic animals and humans. Nevertheless, the evidence should not be generalized to other rodents’ species as the reported prevalence was low, and other sampled rodent species, including *Meriones shawi* and *Mus musculus*, were tested negative in the available study (Selmi et al., 2021a). In addition, associated rodent ectoparasites tested negative for *Bartonella* DNA, which means that the main transmission route in rodent-associated cycles remains unclear. Rodents should therefore be framed as a potential but incompletely characterized wildlife compartment rather than as confirmed reservoirs.

In Tunisian studies, *Bartonella* was detected by different diagnostic tools, including serology, PCR, sequencing and phylogenetic analysis (Znazen et al., 2005; Hammami et al., 2013; Belkhiria et al., 2017; Zouari et al., 2017; Selmi et al., 2020a; Selmi et al., 2021a). Human endocarditis investigations relied on serological and molecular tools, whereas animal and ectoparasite studies used PCR targeting genes such as *ftsZ*, *rpoB*, *gltA*, ITS, *rnpB* and *fur*, followed by sequencing step. This molecular evidence is one of the strengths of the Tunisian literature, particularly for distinguishing classified and non classified zoonotic species (Belkhiria et al., 2017; Zouari et al., 2017; Selmi et al., 2020a). However, PCR sensitivity may vary according to sample type, and some weak positive flea pools could not be fully characterized. Besides, serological tests may be affected by cross-reactivity, thereby limiting the accuracy of pathogen identification (Znazen et al., 2005; Belkhiria et al., 2017; Zouari et al., 2017). Standardized multi-target diagnostic workflows would improve comparability between human, animal and ectoparasite studies.

The Tunisian Bartonella evidence base is scientifically valuable but still incomplete. The most important gap is the lack of integration between human clinical data, animal studies, ectoparasite screening and environmental information. Human data are mostly retrospective and concentrated in Sfax, whereas animal and ectoparasite studies are cross-sectional and often based on convenience sampling (Znazen et al., 2005; Hammami et al., 2013; Belkhiria et al., 2017; Zouari et al., 2017). As a result, no national estimate of the burden of bartonellosis is currently available in Tunisia, and both its true prevalence and regional spatial distribution remain insufficiently characterized.

A second major gap concerns transmission pathways. *B. quintana* dominates Tunisian endocarditis cohorts, but the classical body-louse route was not directly demonstrated in the studied patients, many of whom had rural origin or animal contact (Znazen et al., 2005; Hammami et al., 2013). Similarly, fleas collected from domestic animals carried *Bartonella* DNA, but experimental proof of local flea competence is lacking (Zouari et al., 2017). Dromedary and rodent findings also suggest additional ecological compartments, but their reservoir status and spillover potential remain unresolved (Selmi et al., 2020a; Selmi et al., 2021a).

The Tunisian *Bartonella* profile differs clearly from the tick-centered *Rickettsia* model, as it is shaped mainly by severe human endocarditis, domestic and peri-domestic ectoparasites, stray animals and rural exposure settings. The evidence is strongest for human endocarditis, stray dogs and flea-associated molecular detections, whereas dromedaries and rodents represent emerging but still insufficiently characterized compartments. Future work should prioritize systematic *Bartonella* testing in blood culture-negative endocarditis, prospective multicenter clinical studies, expanded surveys in owned pets, stray animals and domestic animal fleas, targeted investigations in camel-exposed populations, and broader screening of synanthropic rodents and their ectoparasites. The taxon-level evidence for *Bartonella* spp. is summarized in Table 1, while the main critical gaps and surveillance priorities for this genus are presented in Table 2.

### 3.3 Anaplasmataceae

The Tunisian evidence on the *Anaplasmataceae* family is dominated by animal and tick-associated data, with a marked contrast between the relatively rich evidence for *Anaplasma* spp., limited evidence for *Ehrlichia* spp., and absence for other genera. Several *Anaplasma* taxa with confirmed, suspected or emerging zoonotic relevance have been reported in domestic animals, wildlife or ticks, including *Anaplasma phagocytophilum*, *Anaplasma ovis*, *A. phagocytophilum*-like genotypes, *Anaplasma platys*, *A. platys*-like strains and camel-associated strains related to *Candidatus* Anaplasma camelii (Sarih et al., 2005; M’ghirbi et al., 2012; Belkahia et al., 2014; Belkahia et al., 2015; Ben Said et al., 2015; Ben Said et al., 2017a,b; Belkahia et al., 2019; Selmi et al., 2019a; Selmi et al., 2020c; Ben Said et al., 2020; Selmi et al., 2021a; M’ghirbi et al., 2022; Rjeibi et al., 2022). In contrast, evidence for *Ehrlichia* circulation in Tunisia is restricted to historical canine serological studies associated with *E. canis*, sporadic detections of *Ehrlichia*-like DNA in ticks, and recent molecular findings in wildlife. Human data remain scarce and are limited to a few serological observations, while no molecularly confirmed human cases have been reported (Ghorbel et al., 1991; Ghorbel et al., 1994; Sarih et al., 2005; Balti et al., 2021). Consequently, for both genera, the lack of molecular confirmation of human infection represents a major One Health knowledge gap.

*A. phagocytophilum* is globally recognized as the agent of the human granulocytic anaplasmosis. In Tunisia, however, the evidence remains animal- and tick-centered. Serological and/or molecular evidence has been reported in horses, cattle, small ruminants, dromedaries and ticks, especially in humid northern and livestock-associated settings (Sarih et al., 2005; M’ghirbi et al., 2012; Ben Said et al., 2014; M’ghirbi et al., 2016, 2022; Rjeibi et al., 2022). In horses, high serological exposure and PCR-positive animals were reported in northern humid areas, while cattle and small ruminants were also shown to be exposed or infected, although their role as maintenance hosts for zoonotic strains remains unclear (M’ghirbi et al., 2012, 2016, 2022). In dromedaries, serological evidence of exposure was reported, but molecular confirmation was inconsistent or absent in some camel studies, raising the possibility of transient exposure or cross-reactivity with related *Anaplasma* genotypes (Ben Said et al., 2014; Belkahia et al., 2015; Selmi et al., 2019a). Ticks provide an additional ecological signal for *A. phagocytophilum* and related genotypes. *A. phagocytophilum* or closely related DNA has been detected in several tick contexts, including *I. ricinus*, *Rhipicephalus* spp., *Hyalomma* spp. and *Hyalomma aegyptium* collected from tortoises (Sarih et al., 2005; M’ghirbi et al., 2012; Belkahia et al., 2019; Rjeibi et al., 2022). These detections suggest that several tick-host systems may contribute to ecological circulation. However, tick positivity should not be equated with confirmed biological vector competence. The detected DNA may reflect an infected blood meal, transient carriage or true tick infection, and these possibilities require experimental or longitudinal evidence to be distinguished (Belkahia et al., 2019; Selmi et al., 2019a; Rjeibi et al., 2022). Despite the global zoonotic status of *A. phagocytophilum*, no confirmed human granulocytic anaplasmosis cases, human PCR detections or human serosurveys were documented in the reviewed Tunisian corpus (M’ghirbi et al., 2012).

Tunisian studies have also reported unclassified strains described as *A. phagocytophilum*-like 1 and *A. phagocytophilum*-like 2. These genotypes are important because they show that *Anaplasma* diversity in Tunisia is broader than classical species-level categories. Molecular surveys in sheep, goats and cattle detected strains genetically related to *A. phagocytophilum*, whereas specific *A. phagocytophilum* was not confirmed in the tested ruminants (Ben Said et al., 2015; Ben Said et al., 2017b). Additional detections in ticks and wildlife-associated contexts further support their ecological relevance (Belkahia et al., 2019; Selmi et al., 2021a). The major interpretive challenge is that “*A. phagocytophilum*-like” should not be treated as equivalent to zoonotic *A. phagocytophilum*. These strains may be related to known lineages, but their host range, pathogenicity and ability to infect humans remain undetermined, both in Tunisia and globally. They should therefore be described as uncertain zoonotic-relevance strains rather than confirmed human health threats (Ben Said et al., 2018).

*A. platys*, *A. platys*-like strains and camel-associated *Anaplasma* strains represent another unresolved issue. Classical *A. platys* is associated with canine infectious cyclic thrombocytopenia, and Tunisian evidence includes molecular detection in dog-associated ticks or canine-related contexts (Sarih et al., 2005). However, the most original Tunisian contribution concerns *A. platys*-like strains detected in dromedaries and ruminants (Belkahia et al., 2015; Ben Said et al., 2017a; Selmi et al., 2019a). In dromedaries, novel strains genetically related to *A. platys* were reported using molecular and phylogenetic approaches, while later work improved differentiation between classical *A. platys* and camel-associated *A. platys*-like strains using PCR-RFLP and sequencing (Belkahia et al., 2015; Selmi et al., 2019a). *A. platys*-like strains were also detected in cattle, sheep and goats, suggesting a broader host range than initially expected (Ben Said et al., 2017a). Findings related to *A. platys*-like are highly relevant for One Health surveillance because they occur at the interface between domestic animals, dromedaries, ticks and emerging zoonotic uncertainty. However, their human pathogenicity remains unconfirmed, and infected animals were often asymptomatic or not clearly linked to clinical disease (Belkahia et al., 2015; Ben Said et al., 2017a; Selmi et al., 2019b). The term “*Candidatus*” Anaplasma camelii should also be used cautiously. Tunisian camel strains are genetically related to camel-associated *A. platys*-like strains discussed in relation to *Ca.* A. camelii, but the taxonomic status and zoonotic implications of these isolates remain unresolved (Selmi et al., 2019c).

*Anaplasma ovis* is the most extensively documented *Anaplasma* species in Tunisian small ruminants. Molecular studies have reported high prevalence and genetic diversity in sheep and goats from northern and central regions, confirming that *A. ovis* is deeply established in Tunisian small-ruminant systems and represents an important veterinary concern (Belkahia et al., 2014, 2017; Ben Said et al., 2015; ElHamdi et al., 2022; M’ghirbi et al., 2022). Additional molecular characterization based on *msp1a*, *groEL* and *gltA* further confirmed the diversity of Tunisian *A. ovis* isolates from small ruminants, camels and associated ticks, supporting the value of high-resolution typing when comparing strains across hosts and regions (Ben Said et al., 2020). From a One Health perspective, *A. ovis* is important because it is increasingly discussed as a species of suspected zoonotic relevance (Chochlakis et al., 2010). However, its zoonotic role in Tunisia remains inferred rather than demonstrated, since no local human infection has been documented in the reviewed corpus (Ben Said et al., 2018; M’ghirbi et al., 2022). Therefore, the most appropriate interpretation is that *A. ovis* is highly prevalent in animals and may represent a potential exposure signal, but its clinical significance for humans in Tunisia remains unknown. The ecological range of *A. ovis* appears broader than small-ruminant systems alone. *A. ovis* DNA has been detected in Tunisian dromedaries, suggesting that camel-rearing systems may participate in arid-zone transmission cycles (Selmi et al., 2020c). Wildlife-associated detections further complicate the picture. *A. ovis* DNA was reported in wild rodents and in a rat flea pool, suggesting that synanthropic rodents and their ectoparasites may participate in additional ecological interfaces (Selmi et al., 2021a). However, these data should not be overinterpreted as proof of a stable sylvatic cycle or confirmed wildlife reservoir status. Larger longitudinal studies are needed to determine whether rodents maintain *A. ovis* independently or reflect spillover from domestic animal environments.

For *Ehrlichia*, the strongest Tunisian evidence concerns *E. canis* in dogs. Historical seroepidemiological work reported high levels of canine exposure across several Tunisian regions, with seroprevalence ranging from 48.6% to 85.4% depending on the region or dog population studied (Ghorbel et al., 1994). These findings establish canine ehrlichiosis as an important veterinary problem in Tunisia and suggest that the domestic dog-tick interface has long been involved in local *Ehrlichia* ecology (Ghorbel et al., 1994). Nevertheless, most historical canine data are based on indirect immunofluorescence rather than species-specific molecular characterization, and serological cross-reactivity between closely related *Ehrlichia* species can complicate species attribution (Ghorbel et al., 1994).

Human evidence is one of the weakest components of the Tunisian *Ehrlichia* literature. An early preliminary study reported seropositivity to *E. canis* in 9 of 89 patients with fever of unknown origin, suggesting possible exposure to *Ehrlichia* spp. (Ghorbel et al., 1991). Additional serological signals among dog owners have also been mentioned, but such findings do not establish confirmed human infection. This distinction is essential because *E. canis*, *E. chaffeensis* and other *Ehrlichia* species may show serological cross-reactivity, and older IFA/IFI-based studies cannot reliably identify the infecting species without molecular confirmation (Ghorbel et al., 1991, 1994). Based on the reviewed corpus, human exposure to *Ehrlichia* spp. has been suggested by serology, but molecular confirmation has not yet been documented in Tunisia.

Molecular evidence from ticks shows that Tunisian *Ehrlichia* diversity extends beyond classical canine serology. In a North African tick survey, *Ehrlichia*-like DNA was detected in *I. ricinus* and *Hyalomma scupense*, formerly referred to as *Hyalomma detritum*, with sequences related to the *E. chaffeensis*/*E. ruminantium* cluster (Sarih et al., 2005). These findings indicate the presence of uncharacterized *Ehrlichia*-related organisms in Tunisian tick fauna, particularly in northwestern humid environments. However, detection of *Ehrlichia* DNA in ticks does not prove that these tick species are competent biological vectors (Sarih et al., 2005; Balti et al., 2021). The brown dog tick, *Rh. sanguineus* s.l., remains central to the domestic ecology of *E. canis*, and suitability modeling suggests that northern and eastern coastal regions of Tunisia provide favorable conditions for this tick, supporting the relevance of the dog-tick interface in urban and peri-urban environments. However, vector suitability should not be interpreted as pathogen prevalence or transmission intensity without molecular screening of ticks and dogs.

In Tunisian context, the most original recent contribution to the Ehrlichia literature comes from wildlife. Molecular screening reported the detection of *E. ewingii* and Candidatus Ehrlichia shimanensis in North African hedgehogs and their ectoparasites, and E. canis in the common Pipistrelle bat tissues (Balti et al., 2021; Selmi et al., 2021b). These findings expand the recognized diversity of Ehrlichia taxa in Tunisian wildlife and highlight wild vertebrates as potentially important but still poorly characterized ecological compartments. *E. ewingii* is recognized globally as a zoonotic species, whereas Ca. *E. shimanensis* remains an emerging and incompletely characterized taxon (Balti et al., 2021). However, the epidemiological significance of these detections remains uncertain, as the contribution of wildlife hosts to Ehrlichia circulation cannot be established without longitudinal studies evaluating infection dynamics, host competence and transmission pathways.

In contrast to *Anaplasma*, for which ruminants and camels are strongly represented in Tunisia, the *Ehrlichia* evidence in livestock and dromedaries remains weak, indirect or negative. Some tick surveys involving small-ruminant or camel-associated contexts did not detect *Ehrlichia* DNA, suggesting that these compartments may be less central for *Ehrlichia* than for *Anaplasma*, although limited sampling prevents firm conclusions (Sarih et al., 2005). *E. ruminantium* should also be treated cautiously. Although it is a major veterinary pathogen in some African regions, the reviewed Tunisian corpus does not document confirmed infection of Tunisian livestock with *E. ruminantium* (Sarih et al., 2005).

Anaplasmataceae illustrate one of the most important One Health gaps identified in this review. Animal and tick signals are abundant, especially for *A. ovis*, *A. phagocytophilum*, *A. phagocytophilum*-like and *A. platys*-like strains, but the absence of human data prevents any conclusion on the real public health burden. For *Ehrlichia*, the strongest evidence concerns *E. canis* exposure in dogs, tick-associated *Ehrlichia*-like DNA and recent wildlife-associated detections, while human exposure remains serological and unconfirmed. Future work should prioritize targeted screening of febrile patients with tick exposure, molecular comparison of human, dog, livestock, camel, wildlife and tick strains, and high-resolution characterization of *Anaplasma*-like and *Ehrlichia*-like organisms. The taxon-level evidence for *Anaplasma* and *Ehrlichia* spp. is summarized in Table 1, while their main critical gaps and surveillance priorities are presented in Table 2. Their contrasting One Health evidence profiles are also illustrated in Figure 2, which highlights the strong animal-vector signal for *Anaplasma* and the more fragmented canine, tick and wildlife signal for *Ehrlichia*.

### 3.4 *Borrelia* spp.

Compared with *Rickettsia* and *Bartonella*, the evidence on *Borrelia* spp. in Tunisia shows a distinct One Health profile. The available literature supports environmental, vector-associated and animal-associated circulation of both the *B. burgdorferi* s.l. complex and relapsing fever-group *Borrelia*, but the human clinical burden remains insufficiently defined (Younsi et al., 2001, 2005; Bouattour et al., 2010; Ben Said et al., 2016; Selmi et al., 2024a). This makes *Borrelia* a particularly important genus for critical interpretation: Tunisia has clear evidence of enzootic circulation, yet human disease evidence remains mainly serological, fragmented or limited to small clinical reports (Mahfoudhi et al., 2015).

The epidemiology of *Borrelia* in Tunisia encompasses two distinct ecological and clinical systems. The first involves the *B. burgdorferi* s.l. complex, the causative agents of Lyme borreliosis, which has been primarily documented in the humid and sub-humid regions of northern Tunisia (Younsi et al., 2001, 2005; Selmi et al., 2024a). Among the Lyme borreliosis genospecies, *B. lusitaniae* is consistently the most frequently detected in Tunisian *Ixodes* ticks, whereas *B. garinii*, *B. burgdorferi* sensus tricto, and *B. valaisiana* have been reported only sporadically (Younsi et al., 2001, 2005). Although molecular analyses have revealed a high degree of genetic homogeneity among North African *B. lusitaniae* isolates, the pathogenic significance of these strains for human health in Tunisia remains uncertain (Younsi et al., 2005).

The second system comprises relapsing fever borreliae, notably *B. crocidurae*, which has been detected in *Ornithodoros erraticus* ticks collected from rodent burrows in arid and Saharan regions of the country (Bouattour et al., 2010). Additional detections of relapsing fever-group *Borrelia* DNA in ticks parasitizing North African hedgehogs suggest the existence of broader wildlife-tick transmission networks, despite the absence of *Borrelia* DNA in hedgehog tissue samples (Balti et al., 2021). These findings indicate that the *Borrelia* epidemiology extends beyond Lyme borreliosis and includes enzootic cycles of tick-borne relapsing fever in southern arid ecosystems that warrant dedicated surveillance and research efforts. Hard ticks, particularly *I. ricinus*, provide the main vector-associated evidence for the *B. burgdorferi* s.l. complex in Tunisia. Early studies documented *B. lusitaniae* in *I. ricinus* collected from northwestern Tunisia, especially in humid and sub-humid areas such as Béja, Bizerte, Siliana, Ain Draham and Jbelel Jouza (Younsi et al., 2001, 2005). These findings identify the northern humid forest belt as the principal ecological focus for Lyme-group *Borrelia* circulation in Tunisia. More recently, *B. lusitaniae* was detected and genetically identified in questing *I. inopinatus* ticks, suggesting that this tick species may also participate in local Lyme-agent cycles (Selmi et al., 2024a). However, the correct interpretation is that *I. inopinatus* is currently a tick species carrying *B. lusitaniae* DNA and a potential vector, not a biologically confirmed vector. Experimental transmission studies and longitudinal field monitoring are therefore required before its role in human risk can be firmly established.

The tick component of the Tunisian *Borrelia* evidence is mainly represented by *B. crocidurae* in *O. erraticus*. This species was detected in *O. erraticus* collected from rodent burrows in arid and Saharan regions, whereas *O. normandi* tested negative in the available study (Bouattour et al., 2010). This distinction is important because soft tick species should not be treated as epidemiologically equivalent. The *O. erraticus*-*B. crocidurae* association defines a southern transmission cycle that is ecologically distinct from the northern *Ixodes*-*B. burgdorferi* s.l. cycle. However, recent human cases of tick-borne relapsing fever are poorly documented in the available Tunisian corpus, and the disease may be underdiagnosed in rural or Saharan settings. The appropriate conclusion is therefore not that relapsing fever is common in southern Tunisia, but that *B. crocidurae* circulates in infected soft ticks and should be considered in the differential diagnosis of recurrent or unexplained febrile illnesses in exposed populations (Bouattour et al., 2010).

A previous molecular study reported *B. burgdorferi* s.l. DNA in goats, sheep, cattle and camels, with higher detection in goats and sheep than in cattle and camels (Ben Said et al., 2016). These findings suggest exposure or possible involvement in enzootic cycles, but they do not prove that these animals are competent reservoirs able to infect ticks. Livestock should therefore be discussed as exposed hosts or possible maintenance hosts for tick populations rather than confirmed reservoirs of Lyme borreliosis agents. This distinction is important because livestock may support tick abundance and human exposure without necessarily maintaining *Borrelia* transmission.

Wildlife-associated evidence provides an important ecological context for interpreting the apparent discrepancy between the frequent detection of *B. lusitaniae* in Tunisian *Ixodes* ticks and the limited documentation of human Lyme borreliosis. In contrast to classical Lyme borreliosis transmission systems in temperate regions of the Northern Hemisphere, where small mammals and some bird species represent highly competent reservoirs for several human-associated *B. burgdorferi* s.l. genospecies (Radolf et al., 2021), North African Lyme-group *Borrelia* cycles appear to involve a greater contribution of reptilian hosts (Doss et al., 2024). In Tunisia, the lizard *Psammodromus algirus* is a frequent host for immature *I. ricinus* stages, and experimental xenodiagnostic studies demonstrated that this reptile can maintain *B. lusitaniae* infection and infect feeding ticks, supporting its role as a competent reservoir within the Tunisian ecological context (Dsouli et al., 2006). Therefore, the predominance of a reptile-associated transmission cycle may represent one ecological factor contributing to the limited overlap between intense enzootic circulation of *B. lusitaniae* and documented human Lyme borreliosis cases. However, this interpretation should remain cautious because human infection risk is determined by multiple interacting factors, including the pathogenic potential of circulating *Borrelia* genotypes, vector competence, reservoir community composition, tick-host preferences and patterns of human exposure (Mead, 2022).

In Tunisia, the available wildlife data should therefore be interpreted as evidence of a specific ecological maintenance system rather than as direct evidence of human transmission. The association between *P. algirus* and *B. lusitaniae* is particularly relevant because this lizard is abundant in Mediterranean environments and frequently parasitized by immature *Ixodes* ticks (Dsouli et al., 2006). Nevertheless, other wildlife compartments remain insufficiently characterized. In the North African hedgehog study, ticks collected from *A. algirus* carried relapsing fever-group *Borrelia* DNA, whereas hedgehog tissue samples were negative, indicating that hedgehogs should not currently be considered confirmed reservoirs but rather hosts supporting infected ectoparasites (Balti et al., 2021).

Despite this evidence of enzootic circulation, the human epidemiological impact of *Borrelia* infection in Tunisia remains incompletely defined. Earlier investigations reported suspected Lyme borreliosis cases and serological evidence suggesting exposure among individuals from northern forested areas, including forest workers, but these observations did not provide molecular confirmation of active infection (Bouattour et al., 2004). A subsequent clinical report described an atypical Lyme borreliosis-like presentation responding to doxycycline; however, this remained an isolated observation mainly supported by serological findings (Mahfoudhi et al., 2015). Consequently, the current evidence supports the existence of human exposure and possible clinical cases, but does not allow estimation of the true national burden or confirmation of the predominant *Borrelia* genospecies responsible for human infection.

Based on the available corpus, there is no clear evidence of successful PCR or culture confirmation of B. burgdorferi s.l. directly from Tunisian human tissue, blood or cerebrospinal fluid (Bouattour et al., 2004; Mahfoudhi et al., 2015). The most likely conclusion is therefore that suspected human Lyme borreliosis exists in Tunisia, but its true frequency, genospecies attribution and clinical spectrum remain poorly characterized. Similarly, although B. crocidurae is documented in soft ticks from arid and Saharan settings, the contemporary human burden of tick-borne relapsing fever is not well quantified (Bouattour et al., 2010).

The Tunisian *Borrelia* evidence base therefore reveals a major One Health paradox. Environmental and animal-associated circulation is well documented, but human clinical relevance remains poorly defined. The main gap concerns the pathogenicity and clinical impact of *B. lusitaniae*, which dominates the vector evidence but has not been clearly linked to molecularly confirmed human disease in Tunisia (Younsi et al., 2005; Selmi et al., 2024a). A second gap concerns livestock, because molecular detection in goats, sheep, cattle and camels is important but does not prove reservoir competence (Ben Said et al., 2016). A third gap concerns vector competence, especially for *I. inopinatus*, which carries *B. lusitaniae* DNA but requires experimental confirmation as a biological vector (Selmi et al., 2024a). For relapsing fever, the main priority is improved clinical awareness and molecular diagnosis of recurrent fever syndromes in southern rural and Saharan settings, particularly near rodent burrows or soft tick habitats (Bouattour et al., 2010).

Therefore, *Borrelia* evidence in the Tunisian context should be considered as at least two distinct groups: a northern *Ixodes*-Lyme-*Borrelia* group and a southern *Ornithodoros*-relapsing fever-*Borrelia* group. The taxon-level evidence for *Borrelia* spp. is summarized in Table 3, while the main critical gaps and surveillance priorities for this genus are presented in Table 4.

### 3.5 *Coxiella burnetii* and *coxiella*-like organisms

*Coxiella burnetii* occupies a particular position in this review. Unlike classical vector-borne bacteria such as *Rickettsia*, *Borrelia*, *Anaplasma* or *Ehrlichia*, *C. burnetii* is primarily a livestock-associated and environmentally transmitted zoonotic bacterium, responsible for Q fever in humans and coxiellosis in animals (Kaabia and Letaief, 2009; Kaabia and Bahloul, 2021). Its inclusion in a review of vector-associated zoonotic bacteria in Tunisia is justified by repeated arthropod-associated detections and by the historical association of *Coxiella* with tick-screening studies, but its epidemiology should not be interpreted as that of a classical tick-borne pathogen (Selmi et al., 2019b; Balti et al., 2021). The Tunisian evidence indicates that the major One Health relevance of *C. burnetii* lies at the interface between livestock, reproductive products, milk, contaminated environments, occupational exposure and human Q fever, whereas tick-associated detections require cautious interpretation (Rekiki et al., 2005; Barkallah et al., 2018; Selmi et al., 2018).

Livestock represents the most significant evidence compartment for *C. burnetii* in Tunisia. In small ruminants, serological studies have identified *C. burnetii* as one of the infectious agents associated with abortion problems in sheep and goats (Rekiki et al., 2005; Guesmi et al., 2023). Early serological data reported flock-level exposure in small ruminants, while more recent work in organized sheep flocks with a history of abortion confirmed that *C. burnetii* remains relevant in reproductive disorders (Rekiki et al., 2005; Guesmi et al., 2023). However, because some studies targeted flocks with abortion histories, these findings should not be generalized as national baseline prevalence without broader population-based surveys.

Molecular evidence further underscores the veterinary and public health significance of *C. burnetii*. DNA of this bacterium was detected in vaginal swabs and milk from sheep incentral-eastern Tunisia. This confirms that infected animals may shed the bacterium through vaginal secretions and birth products (Barkallah et al., 2018). Nevertheless, molecular detection in milk should be interpreted as evidence of potential food-chain relevance rather than direct proof of milk-borne human transmission in Tunisia, particularly because shedding may be intermittent and exposure routes are often multiple (Barkallah et al., 2018; Selmi et al., 2018).

A serological study reported high *C. burnetii* exposure in Tunisian dromedaries and identified age, seasonality and abortion history as impactful factors related to seropositivity (Selmi et al., 2018). These findings suggest that camels should be incorporated into Q fever surveillance in southern Tunisia, especially because camel husbandry, slaughtering, raw milk consumption and close contact during parturition may create occupational exposure contexts (Selmi et al., 2018). However, direct camel-to-human transmission remains insufficiently documented, and seropositivity indicates exposure rather than active shedding. Therefore, camel-associated *C. burnetii* evidence should be interpreted as an important arid-zone One Health signal requiring further integration with human and environmental data (Selmi et al., 2018; Selmi et al., 2019b).

Human Q fever is documented in Tunisia, but its true burden remains poorly defined. Hospital-based data from Sfax described confirmed cases, with clinical presentations dominated by pneumonia and hepatitis, and a proportion of patients reporting direct livestock contact (Ben Arab et al., 2008). These clinical features make Q fever difficult to recognize when laboratory suspicion is low. The key limitation is that available human data are mainly hospital-based or review-based and do not provide a reliable national incidence estimate (Ben Arab et al., 2008). Moreover, Q fever is considered underestimated in Tunisia, partly because it is not routinely suspected, clinical signs are non-specific and the disease is not systematically integrated into mandatory notification systems (Naoufel and Abdelkarim, 2021).

Ticks and other ectoparasites are relevant to the *Coxiella* discussion, but their role must be interpreted cautiously. *C. burnetii* DNA was detected in *Hy. dromedarii* and *Hy. impeltatum* ticks collected from dromedary camels, whereas camel blood was tested negative by PCR (Selmi et al., 2019b). This suggests tick-associated molecular detection in camel systems, but it does not prove biological tick-borne transmission. Similarly, *C. burnetii* DNA was detected in North African hedgehogs and their ectoparasites, including ticks and fleas, but the reservoir competence of hedgehogs and the transmission role of their arthropods remain unclear (Balti et al., 2021). In the Tunisian context, these findings should therefore be described as arthropod-associated or wildlife-associated molecular detections, not as proof of tick-borne Q fever transmission.

A separate issue concerns *Coxiella*-like organisms and tick endosymbionts. These organisms should not be confused with pathogenic *C. burnetii*, because their zoonotic significance is uncertain and they may represent tick-associated symbiotic bacteria rather than human pathogens (Selmi et al., 2019b; Benyedem et al., 2022). In Tunisian cattle-associated *Hyalomma* ticks, a microbiome study did not detect *Coxiella* endosymbionts and instead reported predominance of other symbionts, including *Francisella*-like organisms (Benyedem et al., 2022). This negative finding is important because it prevents overgeneralization about the presence of *Coxiella*-like organisms in all Tunisian ticks and shows that tick microbiomes may vary according to tick species, host and region.

Wildlife data provide an additional but still exploratory perspective on *Coxiella* ecology in Tunisia. The detection of *C. burnetii* DNA in the North African hedgehog and its associated ectoparasites suggests that peri-domestic wildlife interfaces may contribute to the ecological complexity of *Coxiella* circulation beyond traditional livestock settings (Balti et al., 2021). However, the epidemiological importance of these wildlife-associated findings remains uncertain, and further longitudinal studies are required to clarify the contribution of wild hosts to pathogen maintenance and transmission dynamics. In the current Tunisian context, the main public health relevance of *C. burnetii* remains associated with domestic ruminants, camels, reproductive materials, milk and environmental contamination pathways, which are supported by stronger evidence from livestock-associated studies (Rekiki et al., 2005; Barkallah et al., 2018; Selmi et al., 2018; Guesmi et al., 2023).

Tunisian studies used complementary diagnostic approaches, including complement fixation test, indirect ELISA, indirect immunofluorescence, PCR, real-time PCR, microfluidic PCR, sequencing and next-generation sequencing (Rekiki et al., 2005; Ben Arab et al., 2008; Barkallah et al., 2018; Selmi et al., 2018; Selmi et al., 2019c; Balti et al., 2021; Benyedem et al., 2022; Guesmi et al., 2023). In humans, serology is commonly used. However, the distinction between phase I and phase II antibody responses is important for differentiating acute from chronic Q fever, particularly in suspected endocarditis (Kaabia and Bahloul, 2021). In animals, serology indicates exposure, whereas PCR on milk, vaginal swabs, placentas or abortion material provides stronger evidence of shedding or active infection (Barkallah et al., 2018; Rekiki et al., 2005; Guesmi et al., 2023). The main methodological challenge is therefore the interpretation of the sample type: seropositive animals are not necessarily active shedders, negative blood PCR does not exclude infection, and tick PCR positivity does not prove biological transmission.

Despite substantial evidence, several gaps limit the translation of *C. burnetii* research into surveillance. First, human Q fever is documented but remains underestimated because of non-specific clinical signs, limited routine testing and absence of systematic notification (Ben Arab et al., 2008). Second, many livestock studies are based on flocks with abortion problems or selected farm systems, limiting the ability to estimate national baseline prevalence (Rekiki et al., 2005; Vanderburg et al., 2014; Guesmi et al., 2023). Third, environmental transmission is strongly plausible but insufficiently measured, because direct dust or aerosol sampling is lacking in the reviewed corpus (Selmi et al., 2018). Future studies should therefore combine human clinical surveillance, animal reproductive-sample testing, milk monitoring, environmental sampling and occupational exposure assessment, especially in small-ruminant and camel-rearing systems.

For surveillance purposes, *C. burnetii* should be considered as a major One Health pathogen in Tunisia, but not as a classical vector-borne bacterium. Its public health importance is primarily linked to livestock, birth products, milk, aerosols and contaminated environments, whereas tick-associated detections and *Coxiella*-like organisms should be interpreted cautiously and should not be presented as evidence of confirmed tick-borne Q fever transmission (Selmi et al., 2019b; Balti et al., 2021; Benyedem et al., 2022). The taxon-level evidence for *C. burnetii*, *Coxiella*-like organisms and related tick-associated signals is summarized in Table 3, while the main critical gaps and surveillance priorities are presented in Table 4.

### 3.6 Other vector-associated bacteria

Beyond the major bacterial groups treated separately in this review, recent Tunisian studies have revealed a diverse set of additional bacteria associated with ticks, livestock and vector-related ecological interfaces. These taxa include *Francisella*-like endosymbionts, *M. mitochondrii*, *Rickettsiella* spp., opportunistic genera such as *Staphylococcus*, *Acinetobacter*, *Pseudomonas* and *Corynebacterium*, as well as bacteria specifically investigated but not detected, including *Neoehrlichia mikurensis* and *Spiroplasma ixodetis* (Benyedem et al., 2022; Baede et al., 2024; Kratou et al., 2025b). This group also includes the hemotropic mycoplasma, *M. ovis*, molecularly detected in Tunisian sheep and usually considered as an arthropod-borne pathogen rather than a classical zoonotic agent (Rjeibi et al., 2015). These bacteria are discussed here not because they represent confirmed human zoonoses in Tunisia, but because they illustrate the complexity of vector, livestock and microbiome-associated bacterial signals and their potential relevance for future One Health surveillance.

Most available evidence for tick microbiome-associated taxa is based on molecular detection of bacterial DNA, mainly through 16S rRNA next-generation sequencing, qPCR or conventional PCR, rather than culture, species-level confirmation, animal disease, human infection or proven vector competence (Benyedem et al., 2022; Baede et al., 2024; Kratou et al., 2025b). These taxa should therefore be discussed as vector-associated or arthropod-associated bacteria of uncertain zoonotic significance rather than established human pathogens in Tunisia.

The most prominent bacterial signal in Tunisian *Hyalomma* tick microbiome studies is the genus *Francisella*. In cattle-associated *Hyalomma* ticks, particularly *Hy. scupense*, *Hy. excavatum* and *Hy. marginatum*, *Francisella*-related sequences were among the dominant bacterial components, with high relative abundance in some tick groups (Benyedem et al., 2022). In *Hy. dromedarii* collected from dromedary camels in Kébili, *Francisella* also dominated the bacterial microbiome of Tunisian ticks and differed from the microbiome profile observed in Saudi Arabian ticks (Kratou et al., 2025b). These findings indicate that *Francisella*-like bacteria are core components of several Tunisian *Hyalomma* microbiomes.

However, this result must not be interpreted as evidence of tularemia circulation. The genus *Francisella* includes *F. tularensis*, the confirmed zoonotic agent of tularemia, but the sequences detected in Tunisian tick microbiome studies most likely correspond to *Francisella*-like endosymbionts rather than confirmed pathogenic *F. tularensis* (Benyedem et al., 2022; Kratou et al., 2025b). The reviewed corpus does not provide direct evidence that *F. tularensis* was isolated, molecularly confirmed or linked to human disease in Tunisia. The appropriate conclusion is therefore that ticks harbor abundant *Francisella*-like endosymbionts, while the presence of pathogenic *F. tularensis* remains unproven. Future work should use species-specific PCR, multilocus sequencing or whole-genome sequencing to distinguish *Francisella*-like endosymbionts from pathogenic *F. tularensis* and to assess whether these endosymbionts influence tick fitness or pathogen interactions.

*Midichloria mitochondrii* is another important tick-associated bacterium detected in Tunisia. It was reported in both *Hyalomma* and *Ixodes* ticks, including *I. ricinus* and *I. inopinatus* from northwestern Tunisia (Benyedem et al., 2022; Baede et al., 2024). In adult *Ixodes* ticks collected from humid forest environments, molecular detection rates of *M. mitochondrii* were approximately 35–37.1%, indicating that this endosymbiont is common in Tunisian *Ixodes* populations (Baede et al., 2024). The biological significance of *M. mitochondrii* remains uncertain. As an endosymbiont, it may influence tick physiology, reproduction or microbial community structure, but the reviewed corpus does not demonstrate that it causes disease in humans or animals in Tunisia.

Among the other bacterial taxa, *Rickettsiella* spp. represent an emerging tick-associated signal. *Rickettsiella* DNA was detected in questing *I. ricinus* and *I. inopinatus* ticks from northwestern Tunisia, with reported molecular detection rates around 6.5–7.5% (Baede et al., 2024). This finding suggests that *Rickettsiella* spp. are present in Tunisian *Ixodes* populations and may form part of the bacterial community associated with humid forest tick habitats. However, the available evidence remains limited to molecular detection in ticks. The reviewed corpus does not provide evidence of human infection, animal disease, reservoir hosts, clinical cases or transmission routes for *Rickettsiella* spp. in Tunisia. A more accurate interpretation is therefore that *Rickettsiella* spp. are tick-associated bacteria of uncertain medical significance that warrant further characterization rather than confirmed zoonotic agents.

Microbiome studies of Tunisian ticks also detected opportunistic or environmental bacterial genera such as *Staphylococcus*, *Acinetobacter*, *Pseudomonas*, *Corynebacterium*, *Bacillus*, *Erwinia*, *Serratia* and *Janthinobacterium* (Benyedem et al., 2022; Kratou et al., 2025b). Some of these genera include species of medical or veterinary importance, especially *Staphylococcus* and *Acinetobacter*, which may include opportunistic or antimicrobial-resistant organisms (Kratou et al., 2025b). Nevertheless, these findings should be interpreted as microbiome signals rather than evidence of tick-borne disease. Detection by 16S rRNA sequencing usually does not provide enough resolution to identify pathogenic species or virulence traits, and these bacteria may represent environmental contamination, host skin flora, blood-meal remnants or transient microbial passengers (Benyedem et al., 2022; Kratou et al., 2025b). It would therefore be inappropriate to state that Tunisian ticks transmit *Staphylococcus*, *Acinetobacter* or *Pseudomonas* to humans or animals based only on microbiome detection.

Negative findings remain informative in this group because they help define current surveillance boundaries. *N. mikurensis*, an emerging tick-borne human pathogen in Europe, was specifically investigated in northwestern Tunisian *Ixodes* ticks but was not detected (Baede et al., 2024). *S. ixodetis* was also investigated in the same tick context and was not detected (Baede et al., 2024). These results do not prove absence from Tunisia as a whole, because they are limited to specific tick species, regions, sampling periods and sample sizes, but they provide an important baseline for future surveillance.

The main strength of this corpus is the application of modern molecular tools, especially 16S rRNA next-generation sequencing, qPCR, conventional PCR, sequencing and microbial network analysis (Benyedem et al., 2022; Baede et al., 2024; Kratou et al., 2025b; Rjeibi et al., 2015). The major weakness is that much of the evidence remains descriptive. Genus-level microbiome profiling is often insufficient for distinguishing harmless symbionts from pathogenic species, especially in genera such as *Francisella*, *Staphylococcus* or *Acinetobacter* (Benyedem et al., 2022; Kratou et al., 2025b).

The evidence for other vector-associated bacteria, including tick endosymbionts, microbiome-associated taxa, opportunistic genera, *M. ovis* and informative negative findings, is summarized in Table 3, while related critical gaps and surveillance priorities are presented in Table 4. Their predominantly vector/microbiome- and livestock-centered profile is illustrated in Figure 3, emphasizing that these taxa should be interpreted mainly as ecological, endosymbiotic or veterinary signals rather than confirmed zoonotic threats.

## 4 Discussion: toward an integrated one health surveillance and action framework for Tunisia

This review highlights key challenges for implementing an integrated One Health surveillance framework for vector-borne zoonotic bacteria in Tunisia. The main challenge is no longer pathogen detection, but integrating clinical, veterinary, entomological, wildlife, environmental, and microbiome data to assess epidemiological significance and guide public health action.

### 4.1 Tunisia as a mosaic of One Health systems

The Tunisian evidence should not be interpreted as a uniform national epidemiological pattern. Instead, vector-associated zoonotic bacteria appear to circulate through a mosaic of ecological and socio-economic interfaces that differ in climate, landscape, dominant vectors, animal hosts and human exposure pathways. This interface-based interpretation is summarized in Figure 4, which should be viewed as a conceptual gradient rather than as an administrative map.

**FIGURE 4:**
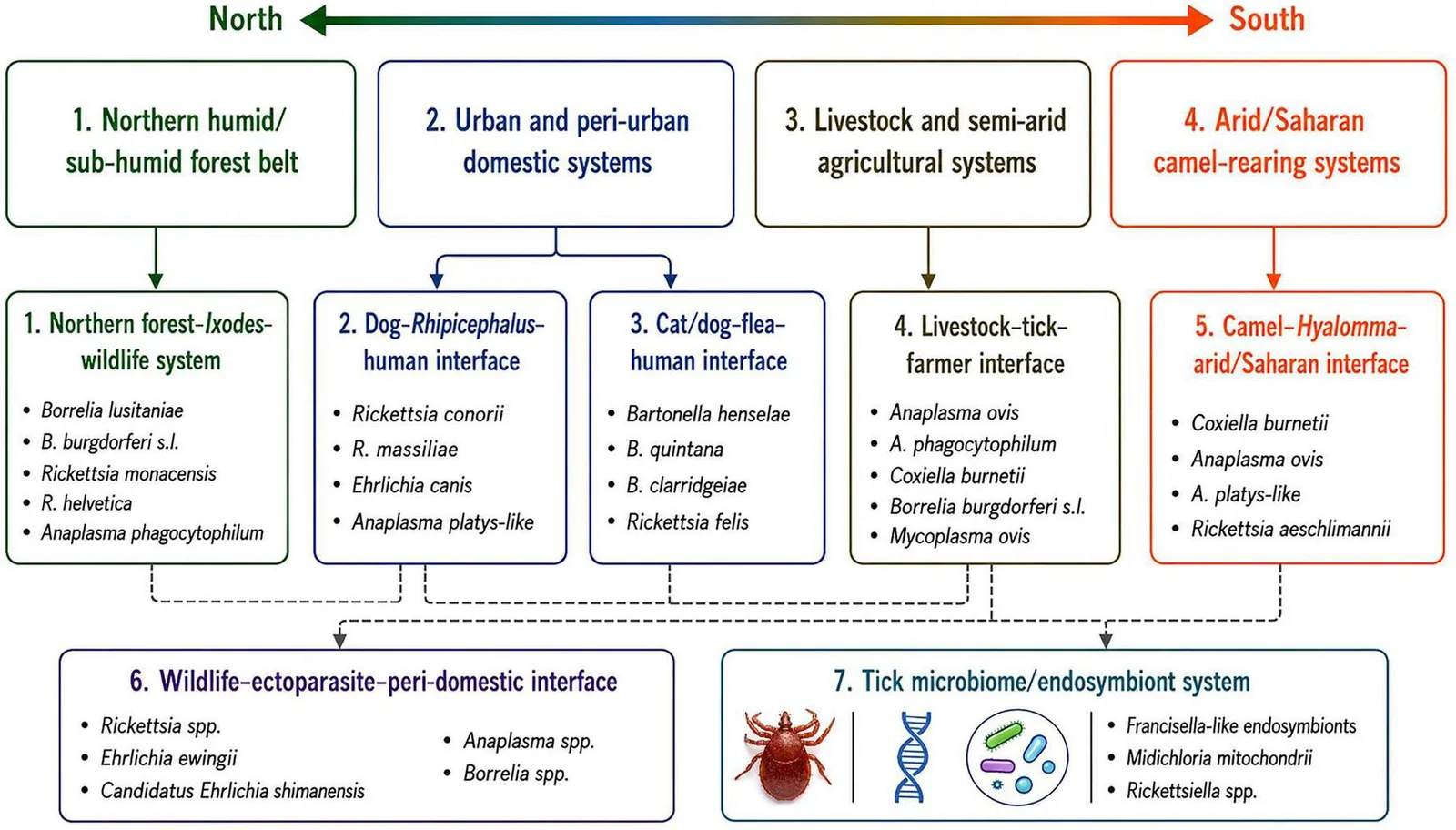
Ecological and socio-epidemiological interfaces of vector-associated zoonotic bacteria in Tunisia. The figure presents a conceptual north-to-south ecological gradient of vector-borne and vector-associated zoonotic bacteria in Tunisia, from northern humid/sub-humid forest systems to arid and Saharan camel-rearing systems. It summarizes the main ecological and socio-epidemiological interfaces identified in this review, including the northern forest-*Ixodes*-wildlife system, dog-*Rhipicephalus*-human interface, cat/dog-flea-human interface, livestock-tick-farmer interface, camel-*Hyalomma*-arid/Saharan interface, wildlife-ectoparasite-peri-domestic interface, and tick microbiome/endosymbiont system. The figure highlights that Tunisian evidence should be interpreted through ecological interfaces and One Health exposure settings rather than through administrative regions alone.

Several major One Health systems can be distinguished. Domestic and peri-domestic settings are mainly shaped by dog-tick-human and cat/dog-flea-human interfaces, which are particularly relevant for *Rickettsia*, *Ehrlichia* and *Bartonella* circulation (Ghorbel et al., 1994; Znazen et al., 2005; Hammami et al., 2013; Znazen et al., 2013; Khrouf et al., 2016; Zouari et al., 2017; Gharbi et al., 2024). Rural livestock systems involve sheep, goats, cattle, horses, ticks, farmers and veterinarians, and are especially important for *Anaplasma*, *Borrelia*, *Rickettsia* and *C. burnetii* (M’ghirbi et al., 2012; Ben Said et al., 2016; Belkahia et al., 2017; Barkallah et al., 2018; Guesmi et al., 2023; Kratou et al., 2023). Arid and Saharan systems are characterized by dromedaries, *Hyalomma* ticks, camel-associated occupational exposure and raw milk practices, with relevance for *Coxiella*, *Anaplasma*, *Rickettsia* and tick microbiome studies (Belkahia et al., 2015; Selmi et al., 2018, 2019a,b, 2020b,c; Kratou et al., 2025b). In northern humid and forested areas, *Ixodes*-associated systems involve wildlife, reptiles and humans exposed during forest-related activities, and are particularly relevant for Lyme-group *Borrelia*, some *Rickettsia* spp., *Anaplasma*-related taxa and tick endosymbionts (Younsi et al., 2001; Sfar et al., 2008; M’ghirbi et al., 2012; Baede et al., 2024; Selmi et al., 2024a; Kratou et al., 2025a). Finally, wildlife and peri-domestic interfaces add further ecological complexity through rodents, hedgehogs, reptiles, wild carnivores and their ectoparasites (Balti et al., 2021; Selmi et al., 2021a,b, 2025; Rjeibi et al., 2022).

Surveillance should therefore be adapted to ecological interfaces rather than administrative regions alone: *Ixodes* surveillance in northern forests; dog, cat, flea and *Rhipicephalus* monitoring in peri-urban areas; integrated animal-tick surveillance in livestock regions; targeted protocols in camel-rearing areas; and systematic monitoring of wildlife interfaces. This provides a practical basis for the proposed One Health surveillance workflow.

### 4.2 Proposed surveillance workflow

An integrated One Health surveillance workflow for Tunisia should follow a stepwise logic that moves from signal detection to targeted sampling, laboratory confirmation, evidence ranking and public health action. This approach is consistent with evidence-mapping and One Health risk-interpretation principles, which emphasize diagnostic certainty, compartmental convergence and cautious interpretation of heterogeneous evidence (Page et al., 2021; Tricco et al., 2018; FAO, WHO, and WOAH, 2019, 2020). The purpose of this workflow is not to multiply isolated screenings, but to connect signals generated in different compartments and interpret them according to their epidemiological relevance. Potential signals may arise from compatible human clinical syndromes, animal or wildlife sentinels, arthropod screening, environmental contamination, food-chain exposure or microbiome datasets. These signals should guide targeted investigations within the relevant ecological interface rather than broad, non-contextual sampling.

Sampling should be tailored to the suspected pathogen and epidemiological interface. Human sampling should prioritize patients with compatible syndromes and relevant exposure histories, while animal and wildlife surveillance should target epidemiologically relevant sentinel hosts. Arthropod surveillance should focus on ticks, fleas, and other ectoparasites across domestic, livestock, camel-associated, forest, and wildlife interfaces (Ghorbel et al., 1994; Znazen et al., 2005; M’ghirbi et al., 2012; Ben Said et al., 2016; Zouari et al., 2017; Balti et al., 2021; Gharbi et al., 2024; Selmi et al., 2024a). For *C. burnetii*, surveillance should extend beyond arthropods and include reproductive materials, milk and environmental samples, because the main risk is livestock-associated and largely aerosol- or environment-based rather than classically vector-borne (Rekiki et al., 2005; Barkallah et al., 2018; Selmi et al., 2018; Guesmi et al., 2023).

Laboratory confirmation should combine serology, PCR/qPCR, sequencing and, when necessary, multilocus typing or whole-genome sequencing. High-resolution molecular tools are particularly important for poorly resolved or emerging taxa, such as *Anaplasma*-like, *Coxiella*-like and *Francisella*-like organisms, as well as for distinguishing closely related bacterial taxa detected in animals, vectors or microbiome datasets (Belkahia et al., 2015; Ben Said et al., 2017a,b; Benyedem et al., 2022; Baede et al., 2024; Kratou et al., 2025a,b). Diagnostic findings should be interpreted within an evidence hierarchy distinguishing exposure, active infection, molecular detection, vector and reservoir competence, and confirmed human disease. This prevents overinterpretation of DNA detection in vectors, animals, or microbiome datasets as evidence of zoonotic transmission. The final step should translate classified evidence into proportionate One Health action. Confirmed or strongly suspected zoonotic signals should trigger clinical awareness, targeted diagnostic testing, veterinary control measures, ectoparasite management, occupational prevention and risk communication. By contrast, microbiome-associated endosymbionts, poorly characterized environmental signals and opportunistic taxa should primarily guide research prioritization and surveillance refinement rather than immediate public health alerts. This proposed workflow is summarized in Figure 5 and is supported by the evidence hierarchy and priority gaps presented in Tables 1–4.

**FIGURE 5:**
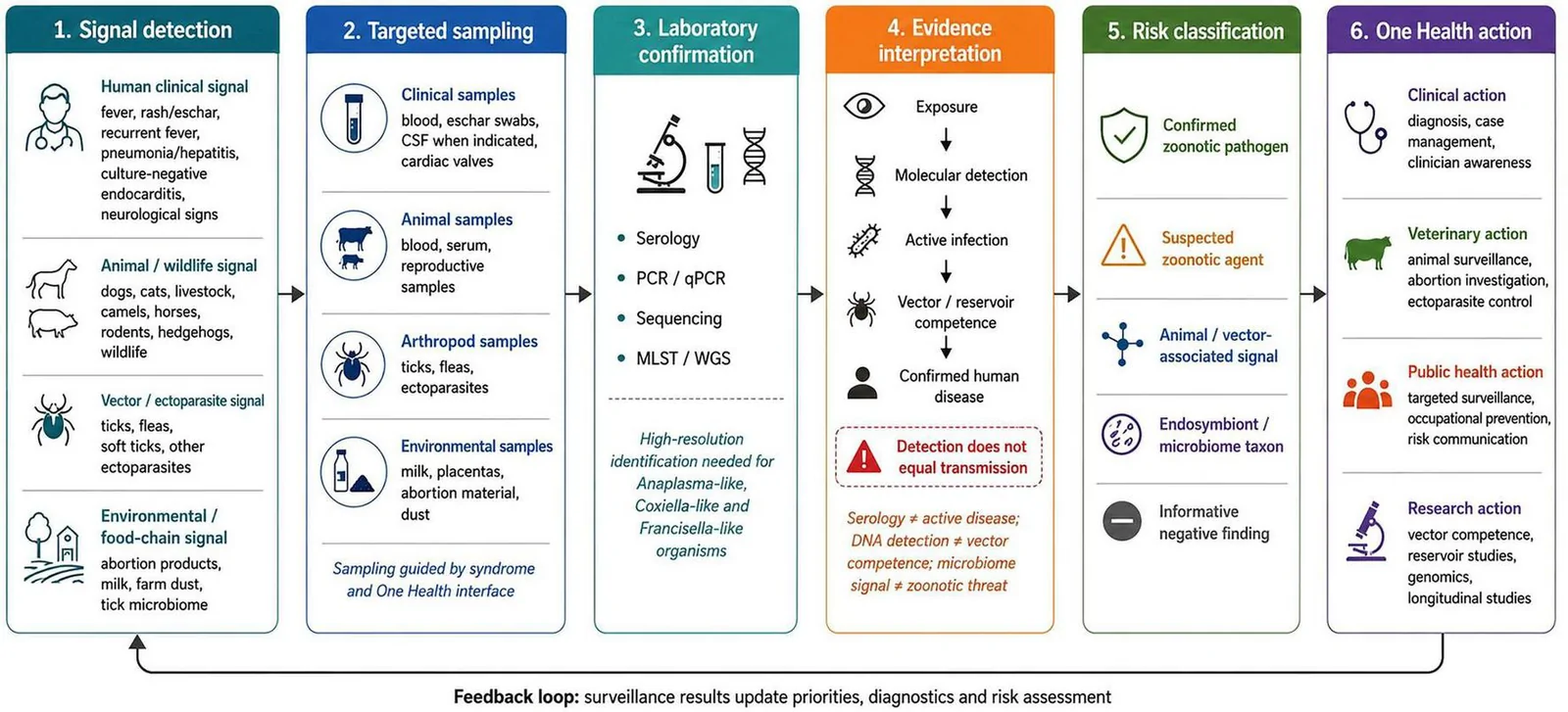
Proposed One Health surveillance framework for vector-associated zoonotic bacteria in Tunisia. The figure presents a stepwise One Health framework for translating human, animal, vector, environmental and microbiome signals into evidence-based surveillance and action. The workflow moves from signal detection to targeted sampling, laboratory confirmation, evidence interpretation, risk classification and One Health action. It emphasizes that molecular detection, exposure, active infection, vector or reservoir competence, and confirmed human disease should be interpreted as distinct levels of evidence. The feedback loop indicates that surveillance outputs should continuously refine sampling priorities, diagnostic strategies and risk assessment.

### 4.3 Strategic recommendations for surveillance, research and public health action

The Tunisian evidence supports a progressive One Health strategy focused on priority interfaces, compatible clinical syndromes and evidence-based risk classification. Human surveillance should prioritize patients showing unexplained fever, rash, eschar, recurrent fever, pneumonia, hepatitis, neurological signs after arthropod exposure, or blood culture-negative endocarditis. Particular attention should be given to exposed groups, including farmers, shepherds, veterinarians, abattoir workers, camel breeders, forest workers, military personnel and people living in close contact with dogs, cats, livestock, camels, rodents or ectoparasites. This targeted approach would improve clinical recognition while avoiding non-specific or poorly contextualized screening. Veterinary and entomological surveillance should be directly connected to human clinical investigation. Dogs, cats, livestock, dromedaries, wildlife and ectoparasites should be used as sentinel compartments according to the ecological interface involved. However, animal or arthropod positivity should not be interpreted in isolation. Each finding should be assessed through an evidence hierarchy that distinguishes exposure, active infection, DNA carriage, vector competence, reservoir competence and confirmed human disease.

Future research should therefore prioritize studies that connect compartments rather than studies that only add isolated detections. Longitudinal investigations, paired human-animal-vector sampling, strain comparison across compartments and systematic reporting of negative findings would substantially improve the Tunisian evidence base. Under-sampled regions, especially central, southern, Saharan and peri-urban settings, should receive greater attention. These areas are particularly important because they include livestock systems, camel-associated environments, peri-domestic exposure and wildlife interfaces that remain incompletely characterized.

A shared national One Health data platform would help transform scattered findings into actionable surveillance. Such a platform should link human cases, animal infections, arthropod detections, wildlife findings, environmental samples, diagnostic methods, geographical information, seasonality and sequence data. Its main value would be to classify evidence according to public health relevance, separating confirmed zoonotic pathogens, suspected zoonotic agents, animal pathogens with uncertain human significance, vector-associated DNA signals, endosymbionts, opportunistic microbiome taxa and informative negative findings. Operationally, this strategy should support proportionate public health action. Confirmed or strongly suspected zoonotic signals should lead to clinical awareness, targeted diagnostics, veterinary control, ectoparasite management, occupational prevention and risk communication. In contrast, weakly characterized microbiome or environmental signals should mainly guide research priorities and surveillance refinement. These practical priorities are aligned with the genus-specific and cross-cutting gaps summarized in Tables 2, 4 and with the surveillance workflow proposed in Figure 5.

### 4.4 Priority research questions

A national One Health strategy for vector-associated zoonotic bacteria in Tunisia should be guided by priority questions that directly connect research, surveillance and public health action. The objective is not only to document additional bacterial detections, but to determine which signals correspond to confirmed zoonotic disease, which remain animal- or vector-associated findings, and which mainly reflect ecological, environmental or microbiome background. Several priority questions emerge from the Tunisian evidence base. First, the true human burden of non-classical rickettsioses, Q fever, bartonellosis, Lyme borreliosis, tick-borne relapsing fever, anaplasmosis and ehrlichiosis remains incompletely defined. Second, the zoonotic relevance of animal-centered taxa such as *A. ovis*, *A. platys*-like strains, *A. phagocytophilum*-like genotypes and camel-associated *Anaplasma* strains requires clarification. Third, the role of specific vectors, including *I. inopinatus*, *Rh. sanguineus*s.l., *Hyalomma* spp., *O. erraticus*, fleas and wildlife-associated ticks, should be evaluated beyond DNA detection. Fourth, the contribution of dromedaries, canids, ruminants, rodents and wildlife to maintenance or spillover cycles remains insufficiently resolved. Finally, the functional role of tick endosymbionts, including *Francisella*-like organisms and *M. mitochondrii*, in vector fitness or pathogen interactions represents an important emerging research direction.

Addressing these questions will require study designs that connect compartments rather than examine them separately. Priority should be given to longitudinal surveillance, paired human-animal-arthropod sampling, molecular comparison of strains across hosts and vectors, environmental sampling in high-risk interfaces and systematic reporting of negative findings. Such approaches would help distinguish incidental detection from epidemiologically meaningful circulation and would improve the interpretation of animal, vector, wildlife and microbiome signals. The added value of this research agenda is that it links unresolved scientific questions to decision-making. A bacterium detected only in a tick microbiome should be classified as an ecological signal requiring characterization, not as an immediate public health threat. A bacterium detected in animals and vectors but not in humans would justify targeted human surveillance in exposed groups. Conversely, a bacterium detected in compatible human disease, animals and arthropods would become a high-priority One Health surveillance target.

## 5 Conclusion

Tunisia has generated substantial evidence on vector-associated zoonotic bacteria, but important gaps remain across bacterial groups, ecological compartments, and levels of epidemiological confirmation. While *Rickettsia* spp., *Bartonella* spp., and *C. burnetii* are the best-documented pathogens, evidence for *Anaplasma*, *Ehrlichia*, and *Borrelia* spp. is derived mainly from animal, vector, and environmental studies, with limited data on human infection. Future progress will depend less on additional detections than on integrating existing evidence within a One Health surveillance framework. Molecular detection should be distinguished from active infection, vector competence, reservoir competence, and confirmed human disease. Strengthening the integration of clinical, veterinary, entomological, wildlife, and environmental surveillance, supported by high-resolution molecular confirmation, will improve risk assessment and public health preparedness. Such an approach could position Tunisia as a regional model for evidence-based surveillance of vector-associated zoonotic bacteria in North Africa and the Mediterranean.
