# Supplementary material for: Vector-borne zoonotic bacteria in Tunisia: from fragmented data to an integrated One Health surveillance framework
Source: Front Microbiol. 2026 Oct 12;17:1951493. doi: 10.3389/fmicb.2026.1951493 (PMC13697242; doi:10.3389/fmicb.2026.1951493)
Supplement: Supplementary file 1 [file Table_1.docx]

Supplementary Table S1. Search strategy, sources and eligibility criteria used for the narrative evidence synthesis.

| Component | Final methodological specification |
| --- | --- |
| Review type | Structured critical narrative review with evidence mapping; no meta-analysis because of heterogeneity in pathogens, hosts, vectors, samples, methods and study designs. |
| Databases searched | PubMed/MEDLINE; Scopus; Web of Science Core Collection; Google Scholar. |
| Publisher/platform searches | ScienceDirect; SpringerLink; Wiley Online Library; Taylor & Francis Online; MDPI; Frontiers. |
| Additional sources | Backward/forward citation checking; local Tunisian or regional journals; conference abstracts; theses; author-held bibliographic files. |
| Last search date | 1 June 2026. |
| Time period covered | No lower date limit; all retrievable records published or available up to 1 June 2026. |
| Languages included | English and French; other languages only when reliable bibliographic and methodological information could be extracted. |
| Core country terms | Tunisia OR Tunisian. |
| Core bacterial/disease terms | Rickettsia; rickettsiosis; Mediterranean spotted fever; *Bartonella*; cat-scratch disease; endocarditis; *Anaplasma*; anaplasmosis; Ehrlichia; ehrlichiosis; *Borrelia*; Lyme; relapsing fever; *Coxiella*; Q fever; *Francisella*; *Midichloria*; *Rickettsiella*; *Mycoplasma ovis*; tick microbiome; endosymbiont. |
| Core vector/host terms | tick(s); flea(s); ectoparasite; arthropod; *Ixodes*; *Rhipicephalus*; *Hyalomma*; *Ornithodoros*; *Ctenocephalides*; camel; dromedary; dog; cat; livestock; small ruminant; cattle; horse; wildlife; rodent; hedgehog; fox, bat. |
| Inclusion criteria | Tunisian evidence on zoonotic, suspected zoonotic, veterinary, vector-associated, wildlife-associated, environmental, food-chain or microbiome-related bacteria. |
| Exclusion criteria | Non-Tunisian studies used only as context; viral/parasitic/fungal/non-target bacterial diseases; inaccessible records without usable information; duplicates; records without usable diagnostic or methodological detail. |
