# Supplementary material for: Vector-borne zoonotic bacteria in Tunisia: from fragmented data to an integrated One Health surveillance framework
Source: Front Microbiol. 2026 Oct 12;17:1951493. doi: 10.3389/fmicb.2026.1951493 (PMC13697242; doi:10.3389/fmicb.2026.1951493)
Supplement: Supplementary file 2 [file Table_2.docx]

Supplementary Table S2. Genus-level screening and inclusion summary.

| Bacterial group | Records screened in genus-specific file | Final evidence retained for synthesis | Main handling notes |
| --- | --- | --- | --- |
| *Rickettsia* spp. | 28 records | 19 unique Tunisian primary studies | Duplicates and non-Tunisian/contextual records were removed or used only as background. |
| *Bartonella* spp. | 15 records | 7 unique directly relevant Tunisian evidence records | Nine directly relevant entries were present, including two duplicates; contextual and non-relevant records were not used for grading. |
| *Anaplasma* spp. | 26 records | 23 directly relevant records after duplicate-aware screening | Duplicated accepted manuscripts/repeated uploads were consolidated in the narrative synthesis; context-only records were not used as primary Tunisian evidence. |
| *Ehrlichia* spp. | 15 records | 10 directly relevant records | Includes veterinary-context records interpreted cautiously; indirect and non-relevant records were excluded from evidence grading. |
| *Borrelia* spp. | 14 records | 9 directly relevant records | Includes human clinical/serological records, tick studies, livestock study, wildlife/vector study and review/context records when relevant. |
| *Coxiella* spp. | 11 records | 11 directly relevant records | Includes human Q fever, livestock/camel serology or molecular studies, tick/wildlife data and *Coxiella*-like/endosymbiont information. |
| Other vector-associated bacteria | 14 records | 4 unique Tunisian evidence records | Mainly microbiome/endosymbiont studies; duplicates, regional reviews and non-target/context records were not used as primary evidence. |
| Total | 123 records | 83 retained records before final duplicate/context interpretation | This number reflects genus-file screening logs; final narrative synthesis consolidates duplicates and interprets context-only records separately. |

Note: Genus-level assignments are not mutually exclusive, as individual studies may report multiple bacterial genera.
