# Supplementary material for: Vector-borne zoonotic bacteria in Tunisia: from fragmented data to an integrated One Health surveillance framework
Source: Front Microbiol. 2026 Oct 12;17:1951493. doi: 10.3389/fmicb.2026.1951493 (PMC13697242; doi:10.3389/fmicb.2026.1951493)
Supplement: Supplementary file 3 [file Table_3.docx]

Supplementary Table S3. Qualitative evidence grading framework used to assign strong, moderate, limited and absent/not documented categories. #

| **Evidence level** | **Decision rule used in this review** | **Typical supporting features** | **Interpretation** |
| --- | --- | --- | --- |
| Strong | High-confidence, reproducible or clinically meaningful Tunisian evidence, supported by robust diagnostics and/or cross-compartment convergence. | Repeated independent reports; PCR/qPCR, sequencing, culture or robust clinical-serological evidence; species/genotype-level confirmation; human disease or strong veterinary/public health relevance; multiple compartments when available. | Established circulation, exposure or disease relevance in Tunisia with high confidence for the assessed compartment. |
| Moderate | Credible but incomplete Tunisian evidence. | One or a few consistent studies; reliable molecular or serological detection; partial taxonomic resolution; one dominant compartment with indirect support from others; restricted geographical or ecological coverage. | Documented circulation, exposure or ecological association, but transmission dynamics, human burden or full One Health cycle remain unresolved. |
| Limited | Preliminary, isolated, indirect or weakly resolved evidence. | Single/isolated detection; small sample size; restricted setting; serology without confirmation; genus-level or microbiome/endosymbiont signal; weak or uncertain taxonomic resolution. | Suggestive signal only; epidemiological significance and zoonotic relevance remain uncertain. |
| Absent / not documented | No relevant Tunisian evidence was found, or only well-defined negative studies were available for the assessed compartment. | No detection in available Tunisian corpus; targeted negative studies; insufficient local data. | Knowledge gap or absence of documented evidence, not definitive absence of the agent from Tunisia. |
