# Supplementary material for: Vector-borne zoonotic bacteria in Tunisia: from fragmented data to an integrated One Health surveillance framework
Source: Front Microbiol. 2026 Oct 12;17:1951493. doi: 10.3389/fmicb.2026.1951493 (PMC13697242; doi:10.3389/fmicb.2026.1951493)
Supplement: Supplementary file 4 [file Table_4.docx]

Supplementary Table S4. Diagnostic weighting and interpretive rules applied during evidence classification.

| Evidence type or rule | Weighting / interpretation in this review | Reason for the decision |
| --- | --- | --- |
| Culture, PCR/qPCR, sequencing, multilocus typing or whole-genome evidence | High weight, especially when linked to species/genotype-level confirmation and compatible host/vector/ecological context. | Provides higher biological and taxonomic certainty than exposure-only data. |
| Human clinical diagnosis supported by molecular confirmation or well-characterized serology | High weight for public health relevance. | Directly supports clinical existence of disease in Tunisia when compatible syndrome and diagnostic interpretation are reliable. |
| Serology in humans | Moderate to high weight only when clinically coherent, repeated, paired/confirmed when available, or supported by additional evidence. | Serology may indicate exposure and may cross-react; clinical context is essential. |
| Serology in animals | Usually moderate or limited unless repeated and supported by molecular or epidemiological evidence. | Animal seropositivity indicates exposure but not necessarily active infection, shedding, reservoir competence or zoonotic transmission. |
| Molecular detection in ticks, fleas or other ectoparasites | Evidence of arthropod association, not proof of vector competence. | DNA detection does not demonstrate biological transmission ability. |
| Molecular detection in animals or wildlife | Evidence of infection/exposure or potential sentinel value, not proof of reservoir competence. | Maintenance and transmission require longitudinal, ecological or experimental confirmation. |
| Microbiome/endosymbiont detection | Ecological or functional signal unless species-level pathogenic identity and clinical/epidemiological linkage are demonstrated. | Prevents overinterpretation of endosymbionts or ambiguous 16S reads as zoonotic pathogens. |
| Negative findings | Informative only when sampling context, target, method and sample size are clear; not generalized beyond studied setting. | Non-detection depends on study design, diagnostic sensitivity and ecological coverage. |
